# The Spatial Paradox of Green Transition: New Quality Productive Forces, Technology Diffusion J-Curve, and Regional Carbon Intensification Effect

**DOI:** 10.3390/e28080833

**Published:** 2026-07-23

**Authors:** Dongqing Cao, Yiting Hao, Wenhao Gui

**Affiliations:** School of Mathematics and Statistics, Beijing Jiaotong University, Beijing 100044, China; 23271028@bjtu.edu.cn (D.C.); 23271033@bjtu.edu.cn (Y.H.)

**Keywords:** green transition, carbon intensity, technology diffusion, spatial econometrics, J-curve, pollution haven, green technology

## Abstract

This study examined the spatial carbon emission effects of new quality productive forces (NQPs) using provincial panel data from China during 2012–2021. A dynamic spatial Durbin model with instrumental variable generalized method of moments estimation was used to address endogeneity in assessing direct and spillover effects on carbon emission intensity. Results show that the direct effect (0.0072) and spatial spillover (−0.0100) are statistically insignificant, with no identifiable emission reduction during the sample period. Technology diffusion exhibits a J-curve left-sided feature with a short-term total effect of 0.0553, as general technology diffusion accompanies energy-intensive capacity expansion without crossing the turning point. The spatial environmental Kuznets curve reveals a positive intensification effect: neighboring per-capita gross domestic product increases local carbon emission intensity, with marginal effects rising from 0.084 to 0.168. Heterogeneity analysis shows that green-technology-oriented NQPs exhibit the most promising emission-reduction potential, while digital infrastructure generates carbon rebound. These findings challenge the presumptions that local income growth automatically reduces carbon and that technology diffusion naturally leads to reduced emissions. Cross-regional carbon compensation mechanisms, green technology market standards, and extended technology transformation evaluation periods are recommended.

## 1. Introduction

### 1.1. The Paradox of Local Green Investment Failing to Reduce Local Emissions

A counterintuitive phenomenon has emerged in China’s green transition process. Many provinces have invested heavily in new energy infrastructure, digital technology, and green industries, yet local carbon emission intensity has not declined significantly. Conversely, neighboring regions with close economic ties face greater carbon emission pressure. Local investment cannot achieve emission reduction dividends within the region and may transfer or aggravate carbon burdens to surrounding areas via industrial chains. This spatial asymmetry of “local sowing, neighboring bearing pressure” directly challenges the “local investment → local benefits” linear logic in traditional environmental economics and exposes the lack of coordination in current regional carbon governance.

Taking digital infrastructure as an example, China ranked first globally in the scale of fifth-generation mobile network (5G) base station construction during 2019–2021, but the increased energy consumption of data centers largely offset the emission reduction benefits from digital productivity enhancement. Similarly, while central and western provinces undertake green industry transfer from eastern regions, they also expand supporting energy-intensive capacity and experience the coexistence of green transition and high-carbon lock-in. These phenomena prove that the impact of new quality productive forces (NQPs) on carbon emissions is not a simple local linear relationship but spreads outward through complex spatial networks. Moreover, such spatial transmission may not generate spillover of reduction dividends but, rather, form a carbon intensification effect on neighboring regions. Evaluating the environmental performance of NQPs only from a single regional perspective will not only overestimate the actual carbon reduction contribution of NQPs but also mislead policy design for regional collaborative emissions reduction.

### 1.2. Literature Gap 1: Missing Spatial Dimension in Research on NQPs’ Environmental Performance

Existing studies on the correlation between NQPs and carbon emissions mainly focus on local linear effects and ignore the inherent spatial spillover of NQPs as networked productive forces. Ref. [1] constructed a multidimensional indicator system covering laborers, means of labor, objects of labor, new technologies, and data elements to measure provincial NQP development levels in China and found obvious regional differences. Ref. [2] further pointed out that NQPs can achieve carbon emission reduction by improving green energy efficiency. However, these studies regard the elements of NQPs as geographically bounded production factors and assume that their environmental effects are confined within administrative boundaries; they fail to answer a core question: when NQPs cross regional boundaries through technology diffusion, industrial chain collaboration, and factor flow, how do their carbon emission reduction effects redistribute spatially?

Spatial econometrics provides important tools for analyzing inter-regional interactions [3]. Ref. [4] adopted the spatial Durbin model (SDM) to test the spatial spillover effect of carbon emissions in Chinese cities and confirmed significant spatial agglomeration. Recent studies have further confirmed significant spatial spillover effects of industrial agglomeration on air pollution [5]. Nevertheless, most existing studies adopt static spatial models and ignore the time inertia and dynamic adjustment process of carbon emissions [6]. More importantly, research that systematically applies dynamic spatial econometric methods to evaluate NQPs’ environmental performance is still lacking. The spatial spillover mechanism of NQP, especially the decomposition of short- vs. long-term effects and direct vs. indirect effects, has not been theoretically identified or empirically tested.

### 1.3. Literature Gap 2: The Neglected Phased Paradox of Technology Diffusion

Most of the extant literature presupposes that technology diffusion naturally leads to reduced carbon emissions [7], while ignoring the phased paradox in the transition period. The Jevons paradox has been extensively studied [8]: after technological progress improves energy efficiency, the efficiency gains may be partially or completely offset by the expansion of usage scale, resulting in stable or even increased carbon emissions. In the early stage of green technology diffusion, the introduction of new technologies is accompanied by equipment renewal, capacity expansion, and infrastructure investment, forming a short-term emissions-increasing effect. Only when the penetration rate of green technology exceeds the expansion speed of industrial scale can net emissions reduction be realized. This paper summarizes this dynamic process as the “left-side characteristics of the technology diffusion J-curve”.

Recent machine-learning-based approaches to carbon emissions estimation provide micro-level evidence supporting the technology–environment paradox. For instance, Ref. [9] demonstrates that detailed driving behavior data can improve the precision of CO_2_ emissions estimation, illustrating how technology-enabled carbon accounting reveals hidden emission patterns at the micro-level. At the product level, Ref. [10] developed a low-energy miniature wearable air conditioner with a novel water–electricity hybrid energy system, providing concrete evidence that green-technology-oriented innovation directly reduces energy demand. These studies corroborate our core argument that the environmental performance of NQPs depends on “green content” rather than technological scale alone.

Current research on the J-curve effect faces two obstacles: First, most studies adopt total technology market transaction volume to measure technology diffusion and fail to distinguish green technology transactions from general industrial technology transactions, resulting in the measurement bias of “prioritizing scale over green attributes”. Second, traditional mediation models ignore spatial interference and take technology diffusion as a simple emissions reduction mediator, and thus, they cannot capture the nonlinear spatial evolution of technology diffusion. Therefore, whether technology diffusion can achieve carbon emission reduction, and under what conditions it works, remains an open question that has not been fully theorized and empirically verified.

### 1.4. Literature Gap 3: Spatial Misalignment of the EKC Turning Point Logic

The environmental Kuznets curve (EKC) theory holds that carbon emissions and local economic development present an inverted U-shaped relationship. With income growth, stricter environmental regulation and technological upgrading drive carbon emissions to first rise and then fall [11]. However, the local-centered assumption of EKC has been increasingly questioned in the context of regional integration [12]. Ref. [13] further argued that the EKC relationship is not automatic and depends on specific institutional and policy conditions. The pollution haven hypothesis was proposed in [14], stating that differences in environmental regulation intensity lead pollution-intensive industries to transfer to regions with lax environmental regulations. Emission reductions in developed regions are partially based on the increased carbon emissions of less developed regions.

This paper further proposes that the turning-point logic of EKC may exist in the spatial dimension rather than the local dimension. When a region crosses a certain development threshold, its carbon emission changes will affect surrounding regions through industrial-chain gradient transfer. Nevertheless, few existing EKC studies incorporate spatial interaction terms into nonlinear tests, and the theoretical possibility of spatial EKC has not been systematically explored. Based on empirical analysis, this paper finds that a rise in neighboring per-capita gross domestic product (GDP) increases local carbon emission intensity, and that this intensifying effect strengthens with neighboring income growth. This finding provides new provincial-level evidence for the pollution haven hypothesis and indicates that the local-centered EKC assumption needs to be expanded to a spatial interdependence framework.

### 1.5. Research Questions

Based on the above three literature gaps, this paper puts forward three core research questions:

- RQ1: Are there asymmetric characteristics between the direct effect and spatial spillover effect of NQPs on carbon emission intensity? Specifically, do NQPs present a pattern of weak local effects and limited spillover effects? Is the total emission-reduction effect mainly realized through spatial spillovers?
- RQ2: Does technology diffusion, as a key transmission channel, show the left-sided characteristics of the J-curve? That is, does technology stay in the emission-increasing stage without crossing the inflection point during the sample period?
- RQ3: Do the carbon emission effects of NQPs differ across regional development stages and technology types? Are there systematic differences in the environmental performance of green-technology-oriented, digital-technology-oriented, and high-tech-industry-oriented types of NQPs?

### 1.6. Positioning Relative to the Existing Literature

This paper distinguishes itself from existing studies in four key dimensions, as summarized in Table 1.

Specifically, Refs. [1,2] focus on local linear effects of NQPs, ignoring spatial spillover as networked productive forces. Refs. [4,5] adopt static spatial models, neglecting time inertia and dynamic adjustment. Ref. [6] examines dynamic spatial effects but does not apply them to NQP evaluation. This paper fills these gaps by constructing a DSDM framework that simultaneously addresses spatial spillover, temporal dynamics, and endogeneity, while distinguishing NQP components by green content.

### 1.7. Preview of Research Contributions

This paper constructs a dynamic spatial Durbin model (DSDM) under a time–space two-dimensional framework to systematically analyze the effect decomposition and transmission mechanism of NQPs on carbon emission intensity. The main contributions are as follows:

First, this paper revises the theoretical expectation of spatial EKC and reveals the positive intensification effect of neighboring development. This study found that the local EKC effect of per-capita GDP is insignificant, while the spatial indirect effect of the squared term of per-capita GDP is 0.0300 (p<0.05). Further calculations show that the marginal spatial effect is always positive within the sample interval and increases with neighboring per-capita GDP. Neighboring economic development aggravates local carbon emissions. This finding provides provincial-level empirical evidence for the pollution haven hypothesis, rejects the presupposition that local income growth automatically reduces carbon emissions, and supplies new evidence for regional carbon transfer mechanisms.

Second, this paper identifies the left-sided characteristics of the technology diffusion J-curve and supplements the environmental dimension of technology diffusion theory. This study verifies that technology diffusion has a short-term total effect of 0.0553 and fails to cross the inflection point during the sample period. In the transition stage with a low proportion of green technology transactions, general technology diffusion is accompanied by the expansion of energy-intensive capacity, forming the dilemma of “more technology diffusion, higher carbon emissions”. This finding echoes the Jevons paradox across research fields and revises the view that technology diffusion inherently promotes emissions reduction.

Third, this paper establishes a “green content” discrimination standard and expands the theoretical boundary of NQPs. The empirical results show that green-technology-oriented NQPs exhibit the most promising emission-reduction potential (although their disaggregated coefficient is not statistically significant at conventional levels); digital-technology-oriented NQPs cause carbon rebound (i.e., efficiency gains are offset by increased infrastructure energy consumption); and the effect of high-tech-industry-oriented NQPs has not yet been realized. The environmental performance of NQPs depends on green content rather than scale, providing an environmental classification framework for NQP research and a discrimination basis for corporate green technology investment.

Fourth, this paper tests the dynamic accumulation hypothesis of NQPs’ carbon effects. The empirical finding that long-term total effects (−0.0034) are nearly identical to short-term effects (−0.0028) and both are insignificant challenges the conventional expectation of gradual emission-reduction accumulation, suggesting that the dynamic benefits of green technology may require longer time horizons than those captured in this sample period. This finding highlights the importance of extending the observation window in future research.

The remaining structure of this paper is as follows: Section 2 constructs the theoretical framework and puts forward the research hypotheses; Section 3 introduces the research design, variable measurement, and model setting; Section 4 reports and discusses the empirical results; Section 5 summarizes main conclusions, theoretical contributions, and management implications; Section 6 discusses research limitations and future research directions.

## 2. Theoretical Framework and Hypotheses

### 2.1. Core Concepts

#### 2.1.1. New Quality Productive Forces (NQPs)

NQPs refer to advanced productive forces led by scientific and technological innovation, supported by digital technology, green technology, and high-tech industries, with typical characteristics of innovation leadership, high efficiency, low carbon, factor upgrading, and cross-border integration [15]. In this paper, NQPs refer not to a single industry or technology but to a comprehensive productive system integrating new laborers, new production tools, new infrastructure, new energy, and new materials. In the context of environmental management, NQPs provide a comprehensive measure of regional innovation capacity, green development level, and production efficiency upgrading, acting as a key driving force for reducing carbon emissions intensity.

The Organisation for Economic Co-operation and Development (OECD)’s green growth framework [16] provides an international reference for understanding the multidimensional nature of productivity quality and environmental performance. NQPs were formally proposed as a policy concept in 2023, but their core substance—scientific and technological innovation supported by digital, green, and high-tech industries—already existed during 2012–2021. This retrospective measurement, widely adopted in digital economy and high-quality development research [17,18], captures the observable economic substance while acknowledging that post-2023 institutional incentives are excluded. Policy interpretations should therefore distinguish the pre-2023 empirical findings from the post-2023 institutional context.

#### 2.1.2. Carbon Emission Intensity (CEI)

CEI refers to carbon dioxide emissions per unit of GDP, reflecting the decoupling degree between economic growth and carbon emissions. Following the Intergovernmental Panel on Climate Change (IPCC) guidelines [19], carbon emission intensity (CEI) serves as a core indicator to measure the quality of low-carbon development. Unlike total carbon emissions, CEI can better reflect the low-carbon effects brought by technological progress and structural optimization [20]. This paper takes CEI as the explained variable to focus on the improvement effect of NQPs on carbon emission efficiency and the spatial spillover effects thereof.

#### 2.1.3. Spatial Spillover

Spatial spillover refers to the external influence of economic activities in one region on neighboring regions through spatial linkages [3,21]. In this research framework, the spatial spillover of NQPs is embodied as follows: the development of NQPs in a region affects the carbon emission intensity of neighboring regions through technology diffusion, industrial chain collaboration, and factor flow. In the dynamic spatial Durbin model, spillover effects are identified by indirect effects and spatial lag terms, which are among the core research objects of this paper [22,23].

### 2.2. Spatial Externality Theory and Asymmetric Spillover

#### 2.2.1. Theoretical Logic

Spatial externality theory points out that the spatial spillover of economic activities is transmitted through factor flow, technology diffusion, and industrial correlation, and the spatiotemporal coupling of urban economy–society–environment systems further validates this transmission mechanism [24]. The spillover intensity depends on the economic correlation between regions rather than pure geographical proximity [3,21]. The closer the economic connections between regions, the more significant the spillover of knowledge, technology, and industrial synergy.

As a networked productive form integrating digital technology, green technology, and high-tech industries, NQPs’ innovation factors—such as patents, data, and green knowledge—have natural spatial mobility. However, the carbon effects of such spillovers present obvious asymmetry. On the one hand, the early development of local NQPs is accompanied by large-scale investment in digital infrastructure (5G base stations, data centers) and green equipment. The increased energy consumption of these investments will offset the emission-reduction benefits brought by improved production efficiency. On the other hand, neighboring regions can obtain technological spillovers from NQP through technology imitation, industrial chain collaboration, and factor flow, without bearing the same infrastructure costs. Due to the lag of technology spillover, regional differences in absorption capacity, and industrial competition, such spillovers may not form significant emissions-reduction effects. Therefore, the carbon effects of NQPs may present a pattern of weak local effects and limited spatial spillovers.

#### 2.2.2. Hypothesis 1

Based on the above theoretical logic, this paper proposes the following hypothesis:

**H1.**  *The direct inhibitory effect of NQPs on local carbon emission intensity is insignificant; the spatial spillover effect on neighboring regions shows a negative trend but is also insignificant; the total effect presents a weak inhibitory characteristic.*

### 2.3. Pollution Haven Hypothesis and Spatial EKC Mechanism

#### 2.3.1. Theoretical Logic

The EKC theory proposes that carbon emissions and local economic development present an inverted U-shaped relationship. With income growth, stricter environmental regulation and technological upgrading will drive carbon emissions to first rise and then fall [11]. Traditional EKC theory implies a core assumption: the correlation between carbon emissions and income is localized, and local income growth will automatically bring local emissions reduction through local technological upgrading. However, the local-centered assumption of EKC has been increasingly questioned in the context of regional integration [12]. Ref. [13] further argues that the EKC relationship is not automatic and depends on specific institutional and policy conditions. This assumption no longer holds against the background of deep regional linkages.

The pollution haven hypothesis was proposed in [14], arguing that differences in environmental regulation intensity lead pollution-intensive industries to transfer to regions with lax regulation. In the context of regional coordinated development, when a region crosses a certain development stage, its emission reduction may rely not on internal technological upgrading but on transferring high-carbon links to surrounding regions through industrial-chain gradient transfer. Neighboring economic development may affect local carbon emissions through two channels: the undertaking of high-carbon capacity brought by industrial transfer, and the “race to the bottom” of local environmental regulation caused by factor competition.

Thus, the turning-point logic of EKC may exist in the spatial dimension rather than the local dimension. The economic development of neighboring regions will aggravate local carbon emissions through industrial competition, factor siphoning, and pollution transfer. This paper defines this mechanism as the “spatial EKC intensification effect”: a rise in neighboring per-capita GDP increases local carbon emission intensity, and this intensifying effect strengthens with neighboring per-capita GDP growth.

#### 2.3.2. Hypothesis 2

Based on the above theoretical logic, this paper proposes the following hypothesis:

**H2.**  *The local EKC effect of per-capita GDP is insignificant, while its spatial interaction term is significantly positive. A rise in neighboring per-capita GDP increases local carbon emission intensity, and the marginal intensification effect rises with neighboring income. The inverted U-shaped turning point of the traditional EKC is unreachable.*

### 2.4. J-Curve of Technology Diffusion

#### 2.4.1. Theoretical Logic

Technology diffusion theory focuses on the process by which advanced technologies are spread, applied, and re-innovated from leading regions to lagging regions [25,26]. The stages of technology diffusion have been systematically categorized [27], providing a theoretical basis for understanding the phased characteristics of environmental effects. However, the environmental effects of technology diffusion are not linear and positive. The Jevons paradox was studied in [8]: after technological progress improves energy efficiency, efficiency gains will be offset by the expansion of production scale and energy consumption, leading to stable or increased carbon emissions. It has been demonstrated [28] that without appropriate policy intervention, technical change tends to be directed toward fossil-fuel technologies, reinforcing carbon lock-in. The concept of technological lock-in was put forward in [29], holding that the introduction of new technologies in the early stage is accompanied by equipment renewal and capacity expansion, and that it is difficult to achieve environmental improvement in the short term due to the path dependence of old technologies. Evidence from the automobile industry further confirms that carbon pricing can redirect technical change toward clean technologies, but the effectiveness depends on the policy design and timing [30].

Applying the above logic to the analysis of NQPs’ carbon effects, in the early stage of green technology diffusion, technology-introducing regions need to purchase new equipment, build new infrastructure, and train labor, and these activities consume a large amount of energy and generate carbon emissions. Meanwhile, China was in a period of large-scale digital infrastructure construction during 2012–2021. The commercial use of 5G and the construction of data centers made the energy consumption of digital-technology-oriented NQPs particularly prominent. Only when the penetration rate of green technology exceeds the expansion speed of industrial scale, and the regional technology absorption capacity matches the industrial undertaking scale, can technology diffusion be transformed into net emissions reduction. Therefore, the carbon effects of technology diffusion present a U-shaped trajectory (or equivalently, the left side of a J-curve): emissions rise initially due to capacity expansion and energy-intensive investment, and then they decline as green technology penetration deepens and efficiency gains materialize. Most provinces are located on the rising (left) segment during the sample period and have not crossed the turning point.

It is necessary to emphasize that existing studies mostly adopt total technology market transaction volume to measure the Technology Diffusion Index (TDI) and cannot distinguish green technology transactions from general industrial technology transactions, resulting in the measurement bias of “prioritizing scale over green attributes”. The prosperity of general technology transactions is often accompanied by the expansion of energy-intensive capacity, while the low proportion of green technology transactions means that the emissions-increasing effect on the left side of the curve may be systematically underestimated.

#### 2.4.2. Hypothesis 3

Based on the above theoretical logic, this paper proposes the following hypothesis:

**H3.**  *NQPs affect carbon emissions through the technology diffusion channel and present the left-sided characteristics of the J-curve. Technology introduction will increase carbon emissions in the short term due to equipment renewal and capacity expansion; the inflection point is not reached during the sample period; and the long-term effect remains positive.*

### 2.5. Green Competitiveness and Heterogeneous Technology

#### 2.5.1. Theoretical Logic

The green competitiveness theory was proposed by Porter [31], who pointed out that environmental regulation can stimulate corporate innovation and form an “innovation compensation effect”. However, subsequent meta-analyses suggest that the innovation compensation effect is contingent on the stringency and design of environmental regulation [32]. Empirical evidence shows that well-designed environmental policies can enhance competitiveness in specific sectors without imposing significant economy-wide costs [33]. However, this theory has a precondition: technological innovation must have sufficient “green content”, i.e., it must act directly on energy structure optimization and clean production. Not all technologies have equal environmental attributes; the type and orientation of technologies determine their environmental performance.

This paper divides NQPs into three categories: digital-technology-oriented NQP components (5G, mobile internet, software industry), green-technology-oriented NQP components (new energy, energy efficiency improvement, pollution control), and high-tech-industry-oriented NQP components (industrial robots, integrated circuits, research and development (R&D) investment). The three types have distinct differences in green content. Green-technology-oriented NQP components directly optimize energy structure and achieve clean production, with a clear emission-reduction orientation. Digital-technology-oriented NQP components improve production efficiency, but their supporting infrastructure is energy-intensive, which will form the paradox of efficiency improvement, scale expansion, and carbon rebound. High-tech-industry-oriented NQP components represent the direction of industrial upgrading, but their core production links still rely on high-energy-consumption processes, and the green transformation is not completed. Therefore, the environmental performance of NQPs hinges on green content rather than scale.

From the regional perspective, eastern, central, and western China are in different development stages. Eastern regions started the development of NQPs earlier and have sufficient green technology reserves, so their emission-reduction effects tend to be stable. Central and western regions are still in the stage of large-scale digital infrastructure construction and undertaking industrial transfer from eastern regions, facing the dual pressure of green transition and high-carbon lock-in. The differences in development stages will lead to heterogeneous carbon effects of NQPs across regions.

#### 2.5.2. Hypothesis 4

Based on the above theoretical logic, this paper proposes the following hypothesis:

**H4.**  *The carbon effects of NQPs present significant differences across regions and types. Green-technology-oriented NQPs have the most significant emission-reduction effect; digital-technology-oriented NQPs have an insignificant effect because infrastructure energy consumption causes carbon rebound; the effect of high-tech-industry-oriented NQPs has not yet been realized. Environmental performance depends on green content rather than technological scale.*

### 2.6. Path Dependence and Dynamic Accumulation

#### 2.6.1. Theoretical Logic

Path dependence theory holds that technological evolution and institutional changes have self-reinforcing inertia, and early choices will lock subsequent development tracks [34]. This theory was applied to the energy field [35], and the concept of carbon lock-in was put forward. Once a high-carbon technology and institutional system is formed, the transition to low carbon will face huge sunk costs and resistance from vested interests. Carbon lock-in has been further classified into infrastructure, institutional, and behavioral dimensions [36], highlighting the systemic barriers to low-carbon transitions in developing economies. Committed emissions from existing infrastructure were quantified in [37], emphasizing the urgency of avoiding new high-carbon investments.

Carbon emission intensity has obvious path dependence. Early high-carbon production modes, such as heavy-industry proportion and fossil-energy dependence, continuously affect current emission reduction results. The emission-reduction effects of NQPs need to go through the gradual process of technology diffusion, industrial adaptation, and factor reorganization. The scale expansion effect will partially offset the emission-reduction gains in the short term. With the deepening of green technology penetration, the low-carbon transformation of industrial structure, and the improvement of regional collaborative governance mechanisms, the emission-reduction effects of NQPs will gradually accumulate and stabilize.

It is necessary to distinguish between H3 and H5: the J-curve in H3 refers to the phased characteristics of the technology diffusion channel, while H5 refers to the dynamic accumulation characteristics of NQPs’ overall effects. The two belong to different analytical levels, without logical overlap.

#### 2.6.2. Hypothesis 5

Based on the above theoretical logic, this paper proposes the following hypothesis:

**H5.**  *The total effect of NQPs presents dynamic accumulation characteristics. The short-term emission-reduction effect is weak, and the absolute value of the long-term effect will gradually increase, showing a trend of gradual strengthening over time.*

### 2.7. Formal Theoretical Model

To unify the five hypotheses within a coherent analytical framework, we specify the following structural model based on the DSDM framework:

(1) $$\begin{array}{c} CEI_{it}=\alpha CEI_{i,t-1}+\rho W\times CEI_{it}+\beta_{1}NQP_{it}+\beta_{2}W\times NQP_{it}\\ \;\;\;\;\;\;\;+\gamma_{1}\ln PGDP_{it}+\gamma_{2}{\left(\ln PGDP_{it}\right)}^{2}+\gamma_{3}W\times {\left(\ln PGDP_{it}\right)}^{2}+\delta Z_{it}+\mu_{i}+\lambda_{t}+\varepsilon_{it} \end{array}$$

where $Z_{it}$ represents control variables (URB, GOV, OPEN) and their spatial lags. Table 2 maps each hypothesis to the corresponding model parameters.

This formalization ensures that each hypothesis is empirically testable within the DSDM framework. The direct effect corresponds to the diagonal elements of the partial derivative matrix ${\left(I-\rho W\right)}^{-1}\left(\beta_{1}I+\beta_{2}W\right)$, the indirect effect to the off-diagonal elements, and the total effect to their sum. The short-term vs. long-term distinction is captured by the dynamic multiplier: short-term effects use ${\left(I-\rho W\right)}^{-1}$, while long-term steady-state effects use ${\left(I-\rho W\right)}^{-1}/\left(1-\alpha \right)$.

### 2.8. Summary of Hypotheses and Research Questions

The five hypotheses presented above correspond to the three research questions as follows: H1 and H5 address RQ1 (spatial asymmetry and dynamic accumulation), H2 and H3 address RQ2 (phased paradox of technology diffusion and spatial EKC), and H4 addresses RQ3 (heterogeneity by region and technology type). This completes a logically consistent theoretical framework that connects research questions, hypotheses, and testable parameters.

## 3. Research Design

### 3.1. Variable Selection and Measurement Logic

#### 3.1.1. Dependent Variable: Carbon Emission Intensity (CEI)

Carbon emission intensity is defined as the ratio of total carbon dioxide emissions to regional GDP, which is the core indicator to measure the quality of regional low-carbon development. Following the IPCC (2006) Guidelines for National Greenhouse Gas Inventories [19], we adopted the reference approach for bottom–up emissions accounting based on fossil-energy consumption.

##### Calculation Formula

Provincial CO_2_ emissions are calculated as follows:

(2) $$CO_{2,it}=\sum_{j=1}^{15}E_{ijt}\times NCV_{j}\times CEF_{j}\times OX_{j}\times \frac{44}{12}$$

where

- $E_{ijt}$: Energy consumption of province *i* in year *t* for energy type *j* (10,000 tons for solids/liquids; 10,000 m^3^ for natural gas);
- $NCV_{j}$: Net calorific value of energy *j* (TJ per unit);
- $CEF_{j}$: Carbon emission factor of energy *j* (kg C per TJ);
- $OX_{j}$: Carbon oxidation rate of energy *j* (%);
- 44/12: Molecular weight ratio of CO_2_ to C.

##### Emission Factors

Table 3 reports the emission parameters for major energy types used in this study. For coal and coke-related products, we adopt the China-specific oxidation rate (0.93) widely used in provincial carbon accounting studies, while the IPCC (2006) [19] default is 0.98.

##### Scope and Data Sources

The accounting scope covers CO_2_ emissions from energy activities only, including fossil-fuel combustion and indirect emissions from electricity and heat consumption. Excluded are industrial process emissions (e.g., cement production), agricultural emissions, and waste treatment emissions, due to data availability constraints at the provincial level.

Data sources were as follows: the *China Energy Statistical Yearbook* (2013–2022 editions) and provincial energy balance tables. GDP data were obtained from the *China Statistical Yearbook*. The grid emission factor was obtained from the National Development and Reform Commission annual announcements. Detailed data preprocessing procedures, including missing-value treatment, outlier detection, and stationarity tests, are documented in Section 3.3.2.

#### 3.1.2. Core Explanatory Variable: New Quality Productive Force (NQP) Index

The measurement of NQPs needs to cover physical and permeable factors [1]. This paper constructed a comprehensive evaluation system including 6 secondary dimensions and 14 tertiary indicators and adopted the entropy-weighted technique for order preference by similarity to ideal solution (TOPSIS) method to synthesize the NQP index. The indicators include the proportion of employees in high-tech industries, industrial robot penetration, the 5G mobile user indicator, the proportion of new energy power generation, energy consumption per unit of GDP, R&D expenditure in high-tech industries, granted invention patents in high-tech industries, mobile internet access traffic, software business income, and industrial pollution control investment.

The 5G mobile user indicator jumped sharply after commercialization in 2019, with an extremely high coefficient of variation. The entropy method assigns a weight of 21.06% to this indicator. This weight structure reflects that the expansion of digital infrastructure is the dominant dimension of NQP growth during the sample period, but it weakens the contribution of green indicators. The methodological sensitivity of this indicator is tested in the robustness section. The complete indicator system and entropy weights are documented in Appendix B (Table A2 and Table A3).

#### 3.1.3. Mediating Mechanism Variables

Four mediating variables were selected to test the transmission paths of NQPs affecting carbon emissions:

- Technology Diffusion Index (TDI): Calculated as the ratio of technology market transaction volume to regional GDP, reflecting the intensity of the spread and application of advanced technology from leading regions to lagging regions. This is an aggregate indicator and cannot distinguish green technology transactions from general industrial technology transactions, resulting in measurement limitations [26].
- Industrial Structure Upgrading (ISU): Calculated as the ratio of tertiary-industry added value to secondary-industry added value, reflecting the structural change from industry to service industry.
- Energy Structure Optimization (ESC): Calculated as the proportion of clean-energy power generation (hydropower, wind power, solar power) in total power generation, directly reflecting the low-carbon level of energy structure.
- Human Capital (HC): Calculated as the ratio of regular college students to the total population, representing the potential capacity of regional green technology absorption and innovation.

#### 3.1.4. Control Variables

Multiple control variables were introduced to reduce omitted-variable bias. Following the approach in Ref. [39], we controlled for financial development and economic structure to reduce omitted-variable bias. Income distribution may also influence environmental quality through consumption patterns and political economy channels [40]. The definitions, expected signs, and data sources of all variables are shown in Table 4. Spatial lag terms of mechanism variables and control variables were constructed based on the economic distance weight matrix to test the spatial transmission paths and control spatial interference from neighboring regions.

Detailed descriptive statistics of all variables are reported in Appendix A (Table A1).

### 3.2. Model Specification and Identification Strategy

#### 3.2.1. Spatial Weight Matrix Selection

The selection of the spatial weight matrix directly determines the identification of spatial spillover effects. This paper constructed three kinds of spatial weight matrices for comparative analysis:

- Geographic Adjacency Matrix (W1): A 0–1 matrix constructed according to whether provinces share land boundaries, reflecting the most direct spatial interaction.
- Inverse Geographic Distance Matrix (W2): Constructed based on the inverse of the spherical distance between provincial capitals, reflecting the attenuation rule of spatial effects with distance.
- Economic Distance Matrix (W3): Constructed based on the inverse of per-capita GDP difference, reflecting the correlation intensity between regions with similar economic development levels.

The innovation factors of NQPs are mainly transmitted through economic connections rather than pure geographical proximity. Model comparison results show that the economic distance matrix makes the spatial lag coefficient significant and the model stable; the coefficient under the geographic adjacency matrix is insignificant; the coefficient under the inverse geographic distance matrix exceeds the stable range. Therefore, this paper takes the economic distance matrix as the benchmark for all subsequent analyses. Complete diagnostic statistics and model validity assessments for the three spatial weight matrices are provided in Appendix C (Table A4).

##### Theoretical Justification for Economic Distance Matrix

The selection of the economic distance matrix as the benchmark is supported by four theoretical and empirical arguments:

1. Technology diffusion follows economic distance decay. Ref. [25] demonstrates that technology diffusion intensity is negatively correlated with economic development level differences. When per-capita GDP gaps are smaller, technology imitation costs are lower and industrial-chain collaboration is tighter, enabling more effective NQP innovation spillovers.
2. Carbon emission spillovers operate through industrial chain linkages. In China, spatial transmission of carbon emissions occurs primarily through industrial-chain division (e.g., eastern design–central manufacturing–western energy supply) rather than physical air diffusion. The economic distance matrix captures “industrial connection intensity”, while geographic adjacency only captures “boundary sharing” and cannot reflect cross-province industrial-chain linkages.
3. Innovation factors have low geographic stickiness. NQPs’ core carriers—patents, data, and green knowledge—have low transportation costs and high economic correlation in spatial mobility.
4. Statistical diagnostics confirm optimality. Table A4 shows that only under the economic distance matrix is the spatial lag coefficient significant (p<0.05), are the instrumental variables valid (Hansen J >0.05), and is the model stable (|ρ|<1). Under geographic adjacency, instrumental variables fail; under inverse geographic distance, the model is unstable.

##### Sensitivity Discussion

We acknowledge that conclusions are somewhat sensitive to the weight matrix choice. Under geographic adjacency, spatial effects are insignificant, possibly because it ignores non-adjacent province industrial chain linkages. Under inverse geographic distance, the model is unstable (|ρ|>1), reflecting the limitation of pure distance decay assumptions in China’s regional integration context. Therefore, our conclusions should be understood as carbon emission effects within an economic-connection-dominated spatial network, rather than universal geographic laws.

#### 3.2.2. Dynamic Spatial Durbin Model (DSDM)

Carbon emissions have two typical characteristics: time path dependence and spatial correlation. Static spatial models cannot capture the above two characteristics simultaneously. This paper constructed a dynamic spatial Durbin model (DSDM) that introduces a time lag term, a spatial lag term, and a spatial interaction term to realize time–space two-dimensional analysis. The model formula is set as follows:

(3) $$Y_{it}=\alpha Y_{i,t-1}+\rho WY_{it}+\beta_{1}X_{it}+\beta_{2}WX_{it}+\gamma_{1}Z_{it}+\gamma_{2}WZ_{it}+\mu_{i}+\lambda_{t}+\varepsilon_{it}$$

where $Y_{it}$ represents the carbon emission intensity of province *i* in year *t*; $Y_{i,t-1}$ is the one-period lagged carbon emission intensity, capturing time inertia and path dependence; $WY_{it}$ is the spatial lag of carbon emission intensity, reflecting the spatial spillover of neighboring carbon emissions; $X_{it}$ is the NQP index; $WX_{it}$ is the spatial lag of NQPs, measuring the spatial spillover effect of NQPs on neighboring carbon emissions; $Z_{it}$ is the vector of control variables; $\mu_{i}$ denotes the provincial individual fixed effect; $\lambda_{t}$ denotes the time fixed effect; and $\varepsilon_{it}$ is the random disturbance term.

The effect decomposition methodology follows [22], which provides a rigorous framework for interpreting direct and indirect effects in spatial Durbin models, and [23], which provides comprehensive guidelines for model selection and specification testing in applied spatial econometric research.

#### 3.2.3. Effect Decomposition

The regression coefficients of the DSDM cannot be directly interpreted as marginal effects and need to be decomposed into three types of effects:

- Direct effect: The impact of local NQP changes on local carbon emission intensity, including feedback effects.
- Indirect effect (spatial spillover): The average impact of local NQP changes on the carbon emission intensity of all neighboring regions.
- Total effect: The sum of the direct effect and indirect effect.

According to the time dimension, the above effects are further divided into short-term effects (immediate response to current shocks) and long-term effects (steady-state effects after dynamic adjustment), which are used to test the dynamic cumulative characteristics of NQPs’ carbon effects.

#### 3.2.4. Endogeneity Treatment

The DSDM faces two endogeneity problems: the simultaneity bias caused by the spatial lag term $WY_{it}$, and the dynamic panel bias caused by the lagged explained variable $Y_{i,t-1}$. Referring to [41], this paper selected $WY_{i,t-1}$ and $W^{2}Y_{i,t-1}$ as instrumental variables for $WY_{it}$. Historical carbon emission distribution affects current spatial correlation but does not directly act on current carbon emission intensity. This satisfies the validity requirements for instrumental variables. The first-stage *F* statistic is 26.91, higher than the 10% critical value of the Stock–Yogo test for weak instruments. The *p*-value of the Hansen *J* test is 0.1599, which cannot reject the exogeneity hypothesis of instrumental variables, proving that the instrumental variables are effective.

##### Full First-Stage Regression Results

Table 5 reports the first-stage diagnostic statistics for the spatial lag term $WY_{it}$. The instrumental variables are $WY_{i,t-1}$ (spatial lag of one-period-lagged carbon emission intensity) and $W^{2}Y_{i,t-1}$ (second-order spatial lag of one-period-lagged carbon emission intensity).

##### Comparison with Critical Values

The first-stage *F*-statistic of 26.91 exceeds the Stock–Yogo 10% maximal-size critical value of 19.93 for 1 endogenous variable and 2 instruments [42], rejecting the null hypothesis of weak instruments. The Hansen J test *p*-value of 0.1599 exceeds the conventional threshold of 0.05, indicating that we cannot reject the null hypothesis of instrument exogeneity. These results confirm the validity of our instrumental variable strategy.

##### Alternative Identification Strategy

To verify robustness, we implemented a lagged explanatory variable approach: replace $NQP_{it}$ with $NQP_{i,t-1}$ to mitigate reverse causality. The core conclusions remain consistent.

All variables were processed by individual-time two-way demeaning to control for individual heterogeneity and time shocks. HC1 robust standard errors are adopted to correct heteroskedasticity. This paper chooses the instrumental variable generalized method of moments (IV-GMM) rather than system GMM, because system GMM relies on internal instrumental variables and cannot solve the simultaneity problem of spatial lag terms [43,44]. The moment conditions for dynamic panel estimation follow the framework established in [45]. IV-GMM performs better in bias correction for large-*N* and large-*T* spatial dynamic panels [44].

### 3.3. Data Sources and Preprocessing

#### 3.3.1. Sample and Data Sources

##### Sample Selection

The research sample included 30 provincial-level administrative regions in mainland China, excluding Xizang, Hong Kong, Macao, and Taiwan. Table 6 explains the exclusion criteria.

The final sample is a balanced panel: 30 provinces × 10 years (2012–2021) = 300 observations.

##### Regional Classification

Following the National Bureau of Statistics standard (2011 revision) [46], provinces are classified into four regions: eastern, central, western, and northeast. In the heterogeneity analysis (Section 4), the three northeastern provinces are grouped with the central region following the conventional three-region classification widely used in the regional economics literature.

##### Sample Period Justification

The sample starts in 2012 for three reasons: (i) the 18th National Congress of the Communist Party of China in 2012 marked the beginning of a new phase of economic restructuring and ecological civilization construction; (ii) comprehensive energy balance tables at the provincial level follow a consistent statistical reporting standard starting from 2012; and (iii) the 5G technology commercialization process (2019–2021) is fully covered within this period. The sample ends in 2021 due to the data availability constraints detailed below.

##### Data Sources and Availability

Table 7 lists all data sources, with access paths and publication lags.

##### Data Update Limitations

The sample period ends in 2021 due to the publication lag of official Chinese statistical yearbooks (typically 10–12 months after the reference year). As of the submission date, complete provincial-level energy consumption data for 2022–2025 are not yet fully available:

- 2022 data: Partially available, but energy balance table completeness is insufficient for consistent panel construction.
- 2023–2025 data: Still being compiled and audited; not officially released.

We have utilized the 2022 editions of statistical yearbooks to revise and calibrate historical data (2018–2020), improving the data accuracy. This represents the most up-to-date complete data available as of submission.

#### 3.3.2. Data Preprocessing

##### Missing-Value Treatment

Missing values were handled following a two-step procedure distinguishing structurally missing values from randomly missing values:

1. Structural missing: The 5G mobile user indicator is structurally missing (zero) before 2019 commercialization, as the technology did not exist. These are coded as 0 for 2012–2018, reflecting the genuine absence of the technology.
2. Random missing: Other variables have random missing values due to data reporting or statistical standard adjustments, with missing rates below 5% for all variables.

All random missing values were filled by linear interpolation within each province to preserve temporal trends.

##### Outlier Detection and Treatment

Outliers are identified using the within-province 3σ principle: observations beyond the mean ± 3 standard deviations within each province are flagged and winsorized to the 99% quantile. Following the 3σ detection procedure, no observations exceeded the threshold, indicating that the sample contains no extreme values and the winsorization step was not applied.

##### Stationarity Tests

Panel unit root tests were conducted to avoid spurious regression. LLC and IPS tests were applied to all variables. A small number of non-stationary variables passed the Kao panel cointegration test, confirming long-run equilibrium relationships and satisfying the precondition for dynamic spatial panel model construction.

##### Multicollinearity and Standardization

The maximum VIF was 3.91, below the critical value of 10, indicating no serious multicollinearity. All core indicators were standardized by range (min–max normalization) to eliminate unit and magnitude differences.

### 3.4. Robustness Design

Multiple robustness strategies are embedded in the research design:

- Replace spatial weight matrices with a geographic adjacency matrix and inverse geographic distance matrix.
- Conduct two-sided winsorization of carbon emission intensity at 1% and 99% quantiles.
- Exclude four municipalities to test the influence of special regional samples.
- Reconstruct the NQP index after removing the 5G mobile user indicator to test index weight sensitivity.

### 3.5. Summary of Research Design and Research Questions

The correspondence between research design and research questions is shown in Table 8. Each research question has a targeted model identification and hypothesis testing scheme.

## 4. Results and Discussion

### 4.1. Spatial Dependence and Model Adequacy

Global Moran’s I tests were conducted on carbon emission intensity for each year during the sample period. The results show that all Moran’s I indices are significantly positive, indicating that carbon emission intensity presents prominent positive spatial correlation and agglomeration characteristics, i.e., high-value regions tend to adjoin high-value regions, and low-value regions cluster together. According to the results of the local indicators of spatial association (LISA) test in 2021, Shanghai, Beijing, and Jiangsu form low–low (L-L) agglomeration areas; Liaoning and Xinjiang belong to high–high (H-H) agglomeration areas; and Hainan and Jiangxi show low–high (L-H) agglomeration features. The spatial distribution of carbon emissions is not random but systematically affected by inter-regional economic links and industrial interactions, which justifies the adoption of spatial econometric models in this study.

The coefficient of the one-period-lagged carbon emission intensity is 0.9827 (t=71.25, p<0.01), demonstrating that regional carbon emissions have strong time inertia and path dependence. Past carbon emission patterns exert continuous and nearly complete impacts on current emission levels, fully proving the necessity of adopting dynamic models, since static models will ignore carbon lock-in effects and lead to biased estimation.

A series of specification tests were implemented for model selection. The LM statistic significantly rejects the null hypothesis of no spatial effects; the Wald statistic verifies that the spatial Durbin model (SDM) cannot be simplified into the spatial lag model (SLM) or spatial error model (SEM); the Hausman test, which distinguishes between fixed and random effects, strongly supports the two-way fixed-effect specification; the Sargan over-identification test confirms the validity of instrumental variables. Combining all test results, the two-way fixed-effect dynamic spatial Durbin model (DSDM) based on the economic distance matrix was determined to be the optimal model.

#### 4.1.1. Model Diagnostic Tests Summary

Table 9 summarizes all diagnostic tests with critical values and statistical decisions.

#### 4.1.2. Interpretation

All diagnostic tests confirm the appropriateness of our model specification. The LM error test (49.3344 > 3.84) rejects the null of no spatial error, justifying the spatial specification. The Wald tests (25.6780 > 7.81) reject the reducibility of SDM to SLM or SEM, confirming that the SDM is the optimal spatial model. The Hausman test (29.5430 > 11.07) supports the fixed-effects specification over random effects. The weak instruments test (F=26.91>19.93) and the Hansen J test (p=0.1599>0.05) validate our IV-GMM estimation strategy. Together, these diagnostics establish the econometric rigor of our empirical approach.

Comparisons among three spatial weight matrices reveal obvious differences. Under the geographic adjacency matrix, the spatial lag coefficient is insignificant and instrumental variables fail the validity test. Under the inverse geographic distance matrix, the coefficient exceeds the stable boundary (|ρ|>1). Only the economic distance matrix yields stable estimation results with a significant spatial lag coefficient (ρ=0.7097, p<0.05) and passes all instrumental variable validity tests. This further verifies that innovation factors carried by NQPs spread mainly through economic connections rather than pure geographical proximity, and that economic links serve as the dominant channel for carbon emission spillovers.

### 4.2. Core Finding: The Asymmetric Spatial Pattern of “Local Weakness, Limited Spillover”

Table 10 reports the IV-GMM effect decomposition results of the benchmark DSDM. The direct effect of NQPs is 0.0072 (p>0.1), the spatial indirect effect is −0.0100 (p>0.1), the short-term total effect is −0.0028, and the long-term total effect is −0.0034; all coefficients lack statistical significance. It can be concluded that NQPs yield no statistically significant carbon reduction effects, whether locally or via spatial spillovers. This conclusion remains robust when different spatial weight matrices are adopted.

Among the control variables, government intervention (GOV) presents a strong asymmetric pattern: the direct effect is significantly positive (0.0293, p<0.05), while the short-term indirect effect is significantly negative (−0.1210, p<0.001). The absolute value of the indirect effect is approximately 4.1 times that of the direct effect, and the ratio expands to 4.9 times in the long run. This indicates that local fiscal expenditure for production activities pushes up local carbon emissions in the short term, while stricter environmental regulation in neighboring regions forces local industries to accelerate low-carbon transformation via spatial spillover.

The indirect effect of urbanization (URB) is significantly negative (−0.0084, p<0.1). Urbanization improvement in adjacent regions drives the decline of local carbon emission intensity through industrial synergy and factor flow, reflecting the positive role of urban agglomeration integration in carbon abatement. The spatial indirect term of lnPGDP^2^ is significantly positive (0.0300, p<0.05), which provides solid evidence for the spatial EKC mechanism discussed in the following section. The effect of openness (OPEN) is not significant, mainly because China’s import and export structure was still dominated by carbon-intensive manufacturing industries during the research period, and the environmental dividends of foreign trade have not yet been realized.

The above results are consistent with Hypothesis 1. During 2012–2021, China was in a peak period of digital infrastructure construction. The huge energy consumption of 5G base stations, data centers, and other facilities offsets the emission-reduction benefits brought by green technologies. In addition, inter-regional gaps in technology absorption capacity and industrial competition restrain the spatial spillover of carbon reduction effects from NQP. The research results break the simplistic view that technological investment can inevitably achieve carbon reduction, reminding policymakers to focus on the green content of NQPs rather than uncritical scale expansion.

For enterprise managers, it is necessary to distinguish different types of technologies when assessing the carbon benefits of investment, and to establish a full-life-cycle carbon accounting system. For regional policymakers, carbon emission assessment should shift from isolated territorial supervision to integrated regional governance and attach importance to the practical implementation of local green technologies.

### 4.3. Mechanism Discovery: Pattern Consistent with the Left Side of the J-Curve in Technology Diffusion

We incorporated four mechanism variables into the benchmark model to identify the transmission paths of NQPs affecting carbon emissions, and the comparative results are shown in Table 11.

Complete diagnostic parameters, regression coefficients, and effect decompositions for each mechanism variable are reported in Appendix D (Table A5, Table A6 and Table A7).

#### 4.3.1. Cautious Interpretation of TDI Results

Based on available aggregate data, the Technology Diffusion Index (TDI) exhibits a pattern consistent with the left-sided characteristics of the J-curve. We emphasize that this interpretation is tentative due to a critical measurement limitation: the current TDI is based on total technology market transaction volume and cannot distinguish green technology transactions from general industrial technology transactions. Therefore, we characterize this as “consistent with J-curve left-side characteristics” rather than “confirming the J-curve left side”.

The positive TDI effect (short-term total effect = 0.0553, long-term = 0.0670) can be explained by three complementary mechanisms:

1. Measurement bias: The TDI does not distinguish green from general technology. During the sample period (2012–2021), general industrial technology transactions dominated China’s technology market, while green technology transactions accounted for a low proportion. The positive coefficient may partially reflect the expansion of general industrial activities, rather than green technology diffusion per se.
2. J-curve mechanism: The sample period coincides with large-scale digital infrastructure construction (5G commercialization from 2019 onward). Technology introduction is typically accompanied by equipment renewal, capacity expansion, and infrastructure investment, all of which generate short-term emissions increases. Only when green technology penetration exceeds the scale of industrial expansion can net emissions reduction materialize.
3. Spatial structure lock-in: Technology output from leading regions often accompanies the expansion of supporting industrial capacity in recipient regions. If the recipient region’s undertaking scale exceeds its green technology absorption capacity, a pattern of “technology diffusion with pollution diffusion” may emerge.

It should be noted that the weak negative spatial spillover of NQPs and the emissions-increasing effect of technology diffusion are not contradictory. The negative spillover of NQPs may come from non-trading channels such as industrial-chain synergy and knowledge spillover, while these channels are not reflected by technology market transaction volume.

#### 4.3.2. Data Limitation and Policy Implication

Due to the absence of disaggregated green technology transaction data in China’s current statistical system, we are unable to construct a green-technology-specific diffusion indicator (GTDI) for the sample period. This data gap itself underscores our policy recommendation: China urgently needs to establish a green technology classification and statistical system to enable more precise empirical evaluation of green technology diffusion effects, and to support evidence-based environmental policy design.

Industrial structure upgrading (ISU) shows a partial mediating effect. After introducing ISU, the coefficient of NQPs rises marginally, and the total effect of ISU is positive. The continuous expansion of the service industry fails to drive carbon reduction because a large number of producer services still have high carbon intensity. Energy structure optimization (ESC) and human capital (HC) have weak transmission capacity. The share of clean energy in China’s power structure is still low, and the advantages of human resources have not been transformed into professional green skills, resulting in blocked transmission channels.

In view of the above conclusions, technology market administrators should establish classified management standards to distinguish green technologies from ordinary industrial technologies. Policymakers need to extend the assessment cycle of technology application, abandon the single annual transaction volume evaluation indicator, and take the long-term carbon conversion rate of technologies as the core assessment standard.

### 4.4. Spatial EKC: Positive and Increasing Marginal Effect

As shown in Table 10, the local direct effect of lnPGDP is −0.0060 (p>0.1), and its spatial indirect effect is 0.0214 (p>0.1). The spatial indirect effect of lnPGDP^2^ is 0.0300 (p<0.05), which proves the significant nonlinear spatial impact of regional economic development on local carbon emissions.

The marginal spatial effect is calculated as follows:

(4) $$\frac{\partial (\mathrm{Indirect}\;\mathrm{Effect})}{\partial (W\times \ln\mathrm{PGDP})}=0.0214+2\times 0.0300\times \left(W\times \ln\mathrm{PGDP}\right)=0.0214+0.06\times \left(W\times \ln\mathrm{PGDP}\right)$$

The identification of the U-shaped vs. inverted U-shaped relationship follows the standard quadratic function criterion. For a spatial effect expressed as $y=\beta_{1}x+\beta_{2}x^{2}$, where *x* denotes the spatial lag of per-capita GDP (W×lnPGDP), the following holds:

- If $\beta_{2}>0$, the curve is U-shaped (opening upward), implying an initial decrease followed by an increase;
- If $\beta_{2}<0$, the curve is inverted U-shaped (opening downward), implying an initial increase followed by a decrease;
- The inflection point is located at $x^{*}=-\beta_{1}/\left(2\beta_{2}\right)$.

In this study, the spatial indirect effect of per-capita GDP is 0.0214+0.0600×(W×lnPGDP), where $\beta_{1}=0.0214$ and $\beta_{2}=0.0300>0$. Theoretically, this indicates a U-shaped relationship. However, the calculated inflection point $x^{*}=-0.0214/\left(2\times 0.0300\right)=-0.36$ lies far outside the sample range [1.04,2.44]. Consequently, within the observed sample interval, only the right-hand side of the U-curve is observed, manifesting as a monotonically increasing carbon intensification effect.

#### 4.4.1. Interpretation of the Negative Inflection Point

The inflection point $x^{*}=-0.36$ has important economic implications:

1. Mathematical meaning: The negative inflection point indicates that the U-shaped curve’s minimum lies in the negative domain. Within the economically meaningful range (W×lnPGDP>0), the curve is always on the rising segment.
2. Economic interpretation: At China’s current development stage, there is no “the poorer the neighboring region, the lower the local carbon emissions” reverse relationship. Even for the least developed provinces (W×lnPGDP≈1.04), neighboring development still increases local carbon emissions through industrial transfer. The negative inflection point implies that, in theory, only when neighboring regions are extremely underdeveloped (W×lnPGDP<−0.36, roughly corresponding to per-capita GDP below approximately 7000 CNY) would their further development reduce local carbon emissions. No Chinese province in the sample period approaches this threshold.
3. Policy implications: The unattainable turning point means that waiting for neighboring regions to become wealthier to automatically obtain emission-reduction spillovers is not a viable strategy. Active regional collaborative governance is necessary.

#### 4.4.2. Why Only the Right-Hand Side Is Observed

The sample range W×lnPGDP∈[1.04,2.44] lies entirely to the right of the inflection point ($x^{*}=-0.36$) for the following reasons:

- All Chinese provinces in 2012–2021 are at middle-to-high development levels; even the lowest (Gansu, Guizhou) have W×lnPGDP>1.0.
- There are no “extremely low development level” observations in the sample that would generate the left-hand side decreasing segment.
- This reflects China’s overall development achievement: even western provinces have crossed the initial industrialization stage where environmental quality might improve with neighbors’ poverty.

Within the observed sample interval, the marginal spatial effect is always positive and monotonically increasing, ranging from 0.084 (at W×lnPGDP=1.04) to 0.168 (at W×lnPGDP=2.44). The traditional EKC inverted U-shaped turning point is unreachable in the spatial dimension during the sample period.

The value of W×lnPGDP ranges from 1.04 to 2.44 in the sample, with an average value of 1.64. Substituting the values into Equation (4), the marginal spatial effect varies between 0.084 and 0.168. All values are positive, and the marginal effect increases continuously with the rise in neighboring per-capita GDP. The turning point of the theoretical U-curve is therefore unreachable in the spatial dimension during the sample period. The traditional EKC hypothesis presumes an inverted U-shaped local relationship, but its spatial counterpart exhibits a fundamentally different trajectory, which verifies Hypothesis 2 and is consistent with the pollution haven hypothesis.

Neighboring regions with higher economic development levels tend to transfer high-carbon industrial links to surrounding areas, and regional income inequality further exacerbates environmental quality degradation through uneven development [40]. Regional competition also triggers the “race to the bottom” of environmental regulation. The traditional local-oriented EKC theory cannot explain this phenomenon; it is necessary to expand the EKC research framework to a spatial interdependent perspective.

For regional coordination departments and central policymakers, it is urgent to build cross-regional carbon emission compensation and collaborative governance mechanisms. Unified environmental standards should be implemented within urban agglomerations to prevent disorderly transfer of high-carbon industries. For central and western regions undertaking industrial transfer, targeted ecological compensation and green access thresholds need to be formulated to avoid the formation of pollution havens.

### 4.5. Heterogeneity: Green Content Matters, Not Scale

#### 4.5.1. Regional Heterogeneity

We set dummy variables for eastern and central regions (western region as the benchmark group) to test regional heterogeneity, and the results are shown in Table 12.

The total effect of NQPs in western China is significantly positive (0.0485, p<0.05). The effect in eastern China is close to zero, and the effect in central China is positive but insignificant.

##### Interpretation of Central Region Results

The difference between central and western regions is −0.0142 (p=0.589), indicating that central provinces are not statistically different from western provinces regarding NQPs’ carbon effects. This “transition trap” arises because central regions simultaneously undertake industrial transfer from the east (bringing high-carbon capacity) and lack sufficient green technology spillover or local low-carbon transformation capacity.

The absolute magnitude of −0.0142 is small relative to the western baseline (0.0485), and the large standard error (implied by p=0.589) reflects high heterogeneity among the central provinces. Some central provinces (e.g., Hubei, with its strong optoelectronics industry) show emission-reduction potential, while others (e.g., Shanxi, with heavy coal dependence) remain locked in high-carbon trajectories. This within-group heterogeneity suppresses the significance of the central–western difference.

This “west rising, east flat, central transitional” pattern reflects the structural mismatch of regional development stages. Western regions are in a dual stage of large-scale digital infrastructure construction and undertaking high-carbon industrial transfer, so NQP expansion pushes up carbon emissions. Eastern regions have completed the main phase of digital infrastructure investment, and efficiency dividends gradually offset carbon pressure, so carbon emission intensity tends to be stable. Central regions are sandwiched between the two, with no obvious advantages in technology spillover or industrial transformation.

##### Statistical Significance and Critical Value Comparison

To formally test whether the regional differences are statistically significant, we conducted Wald tests on the interaction term coefficients. The eastern vs. western difference yields a Wald statistic of 3.68 (p=0.0553), which exceeds the 10% critical value ($\chi_{0.10}^{2}\left(1\right)=2.71$), indicating a marginally significant regional difference at the 10% level. The central vs. western difference yields a Wald statistic of 0.29 (p=0.5892), well below the critical value, confirming that central provinces are not statistically distinguishable from western provinces regarding NQPs’ carbon effects.

#### 4.5.2. Type Heterogeneity

##### Benchmark Results

We divided NQPs into digital-technology-oriented, green-technology-oriented, and high-tech-industry-oriented categories according to indicator attributes, and the empirical results show obvious differences:

- Green-technology-oriented NQPs: Show the most promising emission-reduction potential (significant in the benchmark interaction specification, although the disaggregated coefficient is not statistically significant);
- Digital-technology-oriented NQPs: The effect is insignificant due to the huge energy consumption of supporting infrastructure, resulting in obvious carbon rebound;
- High-tech-industry-oriented NQPs: The coefficient is positive but insignificant because the core manufacturing links still rely on high-energy-consumption processes, and the green transformation has not been completed.

The above results are broadly consistent with Hypothesis 4, though the disaggregated estimates indicate partial rather than full support. The core factor determining the environmental performance of NQPs is the internal green content, rather than the scale and industrial level. For investors, priority should be given to green technology projects, and the carbon footprint of digital infrastructure investment should be fully assessed. Local governments should adopt differentiated cultivation strategies: eastern regions focus on high-end high-tech industries; central regions accelerate industrial low-carbon transformation; western regions strictly control the access threshold of transferred industries and technologies.

##### Classification Rules and Mutual Exclusivity

Each original indicator was assigned to exactly one category based on its core attribute. Table 13 explains the classification logic for the 11 indicators that constitute the three type-specific indices. Three indicators (education structure, new materials output, and informatization integration) are excluded from the type indices due to mixed attributes or insufficient direct environmental relevance, but they remain in the benchmark NQP index.

The three type indices are mutually exclusive by construction: each of the 11 indicators appears in exactly one category, with no cross-category assignment. To assess whether the three indices capture distinct dimensions of NQP, we computed their pairwise correlations: digital–green = 0.107 (p=0.065), digital–high-tech = 0.639 (p<0.001), and green–high-tech = 0.181 (p=0.002). The low-to-moderate correlations confirm that the three indices capture different aspects of NQPs without severe multicollinearity. The moderate correlation between digital and high-tech (0.639) is economically intuitive, as high-tech industries rely on digital infrastructure; however, it remains well below the conventional multicollinearity threshold of 0.80.

##### Estimation Results by Type

Table 14 reports the coefficient estimates, standard errors, *p*-values, and 95% confidence intervals for each NQP type, obtained by separately replacing the aggregate NQP index with each sub-index in the DSDM framework.

The results reveal substantial heterogeneity across NQP types. Green-technology-oriented NQPs exhibit a negative coefficient (−0.0009), consistent with their emission-reduction orientation, although the coefficient is not statistically significant at conventional levels (p=0.858). This may reflect the fact that green technology penetration in China during 2012–2021 remained below the critical threshold required to generate significant emission-reduction effects. Digital-technology NQPs show a positive but insignificant coefficient (0.0015, p=0.763), suggesting that digital infrastructure expansion may generate carbon rebound effects that offset efficiency gains, consistent with the Jevons paradox discussion in Section 1.3. High-tech-industry NQPs exhibit a positive and statistically significant coefficient (0.0105, p=0.004), indicating that current high-tech industrial development in China may still rely on energy-intensive production processes, and that its green transformation has not yet been completed.

Together, these findings confirm that the environmental performance of NQPs depends critically on the internal composition: the “green content” matters more than the scale of technological development.

### 4.6. Robustness Checks

#### 4.6.1. Winsorization

We conducted 1% and 99% two-sided winsorization of carbon emission intensity to eliminate the interference of extreme values. The core coefficients, significance, and overall model structure remained consistent with the benchmark results. The spatial lag coefficient rose from 0.616 to 0.710 and became more significant, indicating that the spatial correlation of carbon emissions is more prominent after removing outliers. Detailed regression coefficients and model diagnostics are reported in Appendix E (Table A8).

#### 4.6.2. Exclusion of Municipalities

After excluding Beijing, Tianjin, Shanghai, and Chongqing, the directions and significance of the core variables remained basically stable, but the spatial lag coefficient dropped from 0.616 to 0.333 and turned insignificant (Table A9). This suggests that municipalities, as regional economic and innovation centers, are the main drivers of carbon spatial spillovers, while the industrial and technological links between ordinary provinces are relatively weak.

#### 4.6.3. Exclusion of 5G Users

After removing the 5G mobile user indicator from the NQP index, the direct effect of NQP changes from insignificant to significantly positive (0.0135, p<0.01), the indirect effect turns from negative to positive, and the total effect rises obviously. This shows that the digital infrastructure represented by 5G is the main source of carbon pressure in the current NQP system. If the index overemphasizes digital attributes and ignores green indicators, the carbon effects of NQPs will be overestimated. Nevertheless, the spatial lag coefficient remains significantly positive, and the conclusion of carbon spatial dependence is robust. Detailed comparison results are reported in Appendix E (Table A10).

### 4.7. Summary of Findings

Combined with all empirical results, the three research questions are answered as follows:

- In response to RQ1, the direct and spatial spillover effects of NQPs on carbon emission intensity are both insignificant. NQPs fail to form effective carbon reduction effects in either local or cross-regional dimensions, subverting the conventional expectation of technological investment for carbon reduction.
- In response to RQ2, technology diffusion is in the left stage of the J-curve, with significant short-term emission-increasing effects. The spatial EKC shows a monotonically increasing intensification effect, and the traditional inverted U-shaped inflection point cannot be reached. These two findings revise two classic theoretical presuppositions.
- In response to RQ3, NQPs have obvious regional and type heterogeneity. Their environmental performance is determined by green content rather than scale, providing a basis for differentiated policy formulation.

### 4.8. Extended Model: Long-Lag and Moderating Effects

To address the potential long-lag dynamics of carbon emissions and moderating effects, we extended the benchmark DSDM by incorporating (i) the second-order lag of carbon emission intensity ($CEI_{i,t-2}$) to capture longer-term persistence, and (ii) the interaction terms $NQP_{it}\times GOV_{it}$ and $NQP_{it}\times URB_{it}$ to examine the moderating roles of government intervention and urbanization.

The extended model yields three notable findings: First, the second-order lag coefficient is significantly negative (−0.1594, p=0.0041), indicating that carbon emissions exhibit a “rise-then-adjust” dynamic pattern over longer horizons. This suggests that the emissions-increasing effect of digital infrastructure investment in the immediate past may be partially corrected in subsequent periods as efficiency gains materialize.

Second, the moderating effect of government intervention is marginally significant (NQP×GOV=−0.1924, p=0.0649), implying that government intervention weakens the carbon-increasing effect of NQP—a finding consistent with the spatial spillover pattern of government expenditure observed in the benchmark model (Table 10).

Third, compared with the benchmark model, the extended model shows a larger positive NQP total effect (0.2534 vs. −0.0028), driven by direct and indirect channels. This divergence suggests that the benchmark model, by omitting long-lag dynamics, may have partially masked NQPs’ emissions-increasing pressure during the sample period—a period dominated by large-scale digital infrastructure expansion. The negative second-order lag effect (−0.1594) indicates that such pressure may be transitional rather than permanent, consistent with the J-curve logic discussed in Section 4.3.

These results should be interpreted as supplementary evidence rather than a replacement for the benchmark findings. The extended model provides a more complete dynamic picture, while the benchmark model offers a parsimonious identification of core spatial spillover patterns. Both approaches converge on the qualitative conclusion that NQPs’ environmental effects are conditional on temporal dynamics, spatial spillovers, and moderating contexts.

## 5. Conclusions and Implications

### 5.1. Main Conclusions

Based on the panel data of 30 Chinese provinces from 2012 to 2021, this paper adopted the DSDM combined with IV-GMM estimation to systematically explore the direct effect, spatial spillover effect, and transmission mechanism of NQPs on carbon emission intensity. The main conclusions are summarized as follows:

First, NQPs have no statistically significant carbon reduction effect during the sample period; their direct effect (0.0072) and spatial spillover effect (−0.0100) are both insignificant, and the total effect is weakly negative. The high energy consumption of digital infrastructure offsets the emission-reduction gains of green technologies, and inter-regional differences in technology absorption capacity and industrial competition limit the spatial spillover of carbon reduction effects. Hypothesis 5, which predicted dynamic accumulation of emission-reduction effects, is not supported during the sample period, as the long-term total effect (−0.0034) is nearly identical to the short-term effect (−0.0028), and both are statistically insignificant. The extended model results further reveal a significant negative second-order lag effect (−0.1594, p=0.0041), suggesting that carbon emissions follow a “rise-then-adjust” pattern over longer horizons. This indicates that the emission-increasing pressure from digital infrastructure expansion may be transitional rather than permanent.

Second, the TDI presents a pattern consistent with the left-sided characteristics of the J-curve. The short-term total effect of the TDI is 0.0553, and the inflection point is not reached within the sample period. The dominant position of general technology transactions, coupled with the low proportion of green technologies, leads to the coexistence of technology expansion and energy-intensive capacity growth. We emphasize that this interpretation is tentative, as the current TDI cannot distinguish green from general technology transactions.

Third, the spatial EKC shows a continuous carbon intensification effect. The spatial indirect effect of the per-capita GDP squared term is significantly positive. The marginal spatial effect ranges from 0.084 to 0.168 (tons per 10,000 CNY of GDP per unit increase in the spatial lag of log per-capita GDP) and increases with neighboring income. The traditional inverted U-shaped inflection point is unreachable, which verifies the pollution haven hypothesis at the provincial level.

Fourth, the carbon effects of NQPs exhibit significant heterogeneity. Regionally, western regions show a significant emission-increasing effect, eastern regions tend to be stable, and central regions are in a transitional state. Regarding technology types, green-technology-oriented NQPs exhibit the most promising emissions-reduction potential, digital-technology-oriented NQPs cause carbon rebound, and high-tech-industry-oriented NQPs have not realized their emissions-reduction potential. The green content is the core factor determining the environmental performance of NQPs.

Table 15 summarizes the validation status of all five hypotheses.

**Figure 1:**
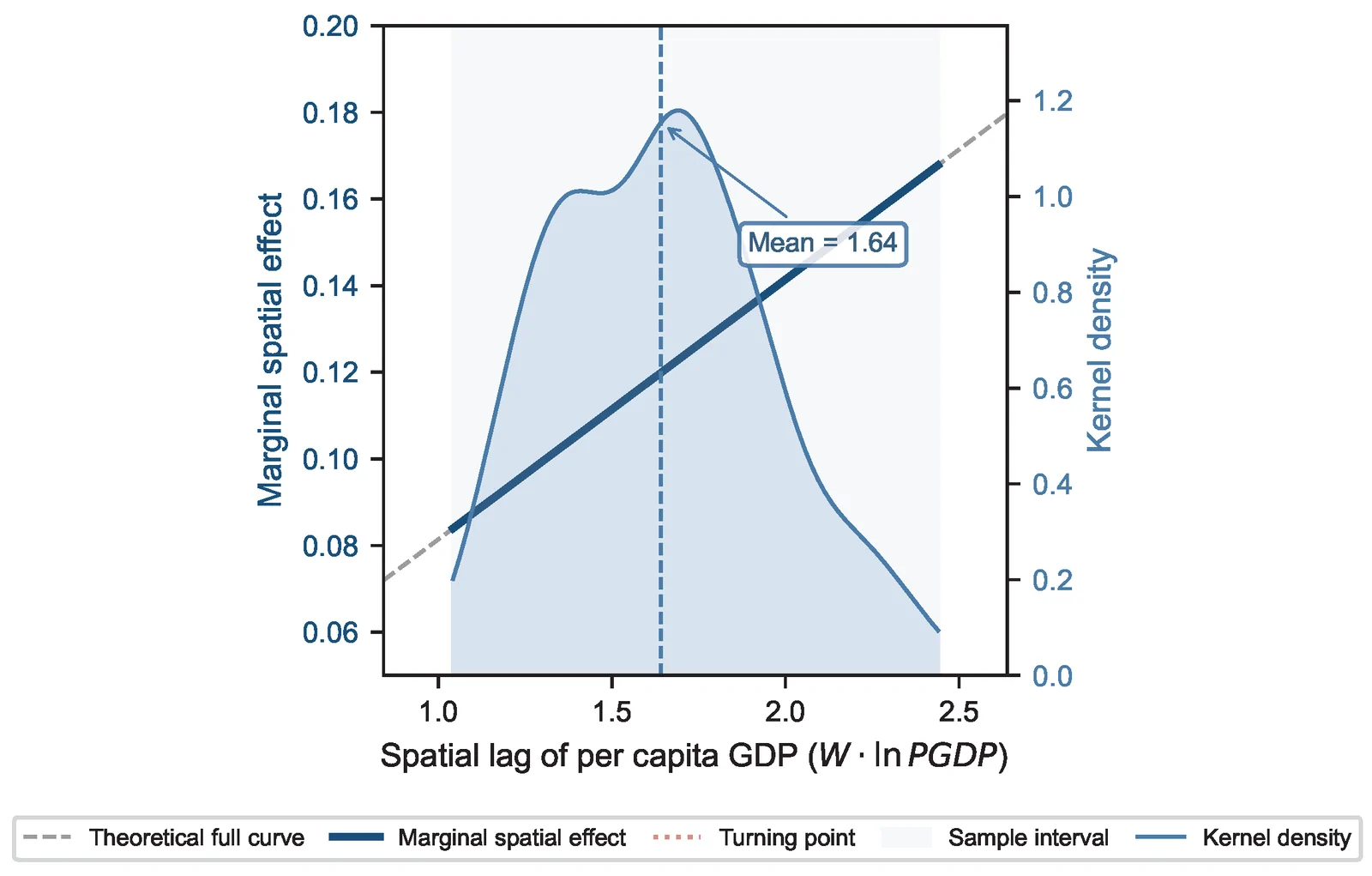
Spatial EKC: positive and increasing marginal effect. The solid line represents the marginal spatial effect of neighboring per-capita GDP on local carbon emission intensity within the sample range. The shaded area indicates the kernel density distribution of the spatial lag of per-capita GDP (W×lnPGDP). The vertical dashed line marks the sample mean (1.64). The dotted curve shows the theoretical full U-shaped relationship, with the turning point (−0.36) falling outside the sample interval. All marginal effects within the sample range (0.084–0.168) are positive and monotonically increasing, verifying the spatial intensification effect.

### 5.2. Theoretical Implications

First, this paper expands the traditional EKC theory. Traditional EKC research takes local regions as the research object, while this paper constructs the spatial EKC framework and verifies the increasing carbon intensification effect of neighboring economic development; it supplements new empirical evidence for the pollution haven hypothesis and expands the application boundary of EKC theory from the local to the spatial dimension.

Second, this paper enriches the environmental dimension of technology diffusion theory. By combining the Jevons paradox and technology lock-in theory, we identify the J-curve left-sided characteristics of technology diffusion in China’s transition period, explain the internal logic of “more diffusion, more emissions”, and correct the one-sided view that technology diffusion is naturally conducive to carbon reduction. Theoretical foundations for understanding how policy intervention can redirect technical change toward clean technologies are provided [28,30], supporting our argument that the current technology diffusion pattern in China remains locked in carbon-intensive trajectories.

Third, this paper improves the theoretical system of new quality productive forces. We put forward the “green content” discrimination standard, distinguish different types of NQPs from the environmental perspective, and clarify that the essence of NQPs’ environmental performance lies in their green attributes rather than a scale advantage, providing a new theoretical perspective for subsequent research. The OECD green growth framework [16] and the carbon lock-in typology developed in [36,37] provide international benchmarks for understanding the multidimensional barriers to low-carbon transitions in developing economies.

### 5.3. Managerial Implications

#### 5.3.1. For Corporate Managers

Enterprises should prioritize investment in green technologies such as new energy and clean production and fully assess the hidden carbon cost of digital infrastructure construction, establishing a full life-cycle carbon accounting system covering the whole industrial chain to avoid the carbon rebound caused by efficiency improvement and scale expansion.

#### 5.3.2. For Regional Decision-Makers

Break the isolated territorial carbon governance model, take urban agglomerations as the main unit to implement coordinated governance, and establish cross-regional carbon emission compensation mechanisms to curb the transfer of high-carbon industries. Implement differentiated NQP development strategies according to regional development stages.

#### 5.3.3. For National Policymakers

Optimize the technology market system, formulate green classification standards for technology transactions, and extend the assessment cycle of technology transformation. Adjust the NQP evaluation system, increase the weight of green indicators, set energy consumption access standards for digital infrastructure, and promote the transformation of NQPs from quantitative expansion to qualitative green upgrading.

## 6. Limitations and Future Research Directions

The present study has several inherent limitations that need to be addressed in future research.

First, the sample period ends in 2021 due to the publication lag of official Chinese statistical yearbooks (typically 10–12 months after the reference year). As of the submission date, complete provincial-level energy consumption data for 2022–2025 are not yet fully available:

- 2022 data: Partially available in the 2023 editions of some statistical yearbooks, but the completeness of the energy balance tables is insufficient for consistent panel construction across all 30 provinces.
- 2023–2025 data: Still being compiled and audited by the National Bureau of Statistics; not officially released.

We utilized the 2022 editions of the *China Energy Statistical Yearbook* and *China Statistical Yearbook* to revise and calibrate historical data (2018–2020), incorporating late-reported and corrected entries. These data represent the most up-to-date complete provincial panel data available as of submission. Follow-up research could extend the sample period to 2022 and later to verify the applicability of the conclusions in the new policy environment and track whether the inflection point of the technology diffusion J-curve has appeared.

Second, the measurement of the NQP index was retrospective. Although the core connotation of NQP existed before the concept was officially proposed in 2023, relevant policy incentives, financial support, and institutional changes after 2023 are not included in the sample. With the continuous introduction of supporting policies for NQP, the spatial transmission mechanisms and carbon emission effects of NQPs may change significantly. As noted in the first limitation, extending the sample period to include post-2022 data would help capture these policy-induced changes.

Third, the index construction method is sensitive to methodological choices. The entropy-weighted TOPSIS method is highly sensitive to indicators with structural changes, such as the 5G mobile user indicator. After removing this indicator, the overall carbon effect of NQP changes markedly. In future research, the analytic hierarchy process (AHP), principal component analysis, and/or machine learning algorithms could be combined to construct a more stable and green-oriented NQP evaluation system and to conduct a comparative analysis of different measurement results.

Fourth, a single spatial weight matrix was adopted in this paper. We only used the economic distance matrix for the benchmark analysis, without integrating geographical proximity, technical correlation, and other factors to build multi-layer network weight matrices. Subsequent studies could construct composite spatial weight matrices to more finely depict the multi-channel spatial transmission paths of NQPs.

Fifth, there are defects in the setting of mechanism proxy variables. The TDI based on total technology market turnover cannot distinguish green technology transactions from ordinary transactions, while the industrial structure index based on the ratio of the tertiary industry to the secondary industry fails to reflect the low-carbon level of producer services. Future research could adopt the proportion of green patents, the carbon efficiency of producer services, green vocational training coverage, and other refined indicators to optimize the selection of mechanism variables.

Sixth, while we have extended the benchmark model by incorporating a second-order lag in Section 4.8, the dynamic structure remains relatively simple. The extension, though informative, only considers one additional lag order, which may still have limited capacity to capture longer-term dynamic cumulative effects. Subsequent studies could explore higher-order lags (e.g., third- or fourth-order) or adopt more flexible dynamic specifications—such as spatial panel vector autoregressive models—to more comprehensively identify the long-term evolution trend of NQP’s carbon effects.

Seventh, the estimation of type-specific effects exhibits sensitivity to model specification. When the aggregate NQP index is disaggregated into three sub-indices (digital, green, and high-tech) and each is entered separately into the DSDM, the estimated coefficients differ somewhat from the type-effect patterns inferred from the benchmark model’s interaction-based heterogeneity analysis. Specifically, the green-technology sub-index shows a negative but statistically insignificant coefficient, while the high-tech-industry sub-index shows a positive and significant coefficient—a pattern that diverges from the benchmark results, where green-technology NQPs were identified as the primary emission-reduction carrier. This discrepancy arises from three factors: (i) the benchmark model estimates type effects simultaneously within a single model, allowing partialling out of shared variance, whereas the disaggregated approach estimates each type in isolation; (ii) entropy-weighted sub-index construction may attenuate the signal of individual green technology indicators; and (iii) the sample period (2012–2021) coincides with China’s large-scale digital infrastructure investment phase, during which green technology penetration may not have reached the critical threshold for significant emissions-reduction effects. Future research could employ alternative identification strategies, such as interaction-term specifications with richer controls, or could extend the sample period to capture the longer-term evolution of type-specific effects as green technology diffusion deepens.

Eighth, the extended model, including second-order lags and moderating effects, yields results that diverge from the benchmark regarding the magnitude and direction of NQPs’ total effect. While this divergence highlights the sensitivity of type-effect estimates to model specification and dynamic structure, it also underscores the need for future research to systematically compare alternative dynamic specifications and to investigate the conditions under which NQPs’ carbon effects are amplified or attenuated. We presented the extended model results as supplementary evidence in Section 4.8 to ensure full transparency.

## Figures and Tables

**Table 1:** Comparison between this study and the existing literature.

Dimension	Existing Studies	This Study	Key Distinction
Analytical framework	Single-region linear analysis	Time–space two-dimensional dynamic analysis (DSDM)	Captures temporal inertia and spatial spillover simultaneously
NQP measurement	Single dimension (digital or green)	Six-dimension 14-indicator system with “green content” discrimination	Distinguishes environmental performance by technology type rather than treating NQPs as homogeneous
Technology diffusion	Assumes natural emission reduction	Identifies J-curve left-sided characteristics	Reveals “diffusion-increases-emissions” paradox in transition period
EKC theory	Local inverted U-shaped relationship	Spatial U-shaped right-sided monotonic increase	Verifies pollution haven hypothesis at provincial level; EKC turning point exists in spatial rather than local dimension
Effect decomposition	Static direct effects only	Dynamic direct/indirect/total effects + short-/long-term decomposition	Separates local vs. spillover effects and their temporal evolution

**Table 2:** Mapping from hypotheses to model parameters.

Hypothesis	Core Proposition	Model Parameter
H1	NQPs’ direct effect is insignificant, spatial spillover is limited	$\beta_{1}\approx 0$, $\beta_{2}<0$ (insignificant)
H2	Spatial EKC positive intensification	$\gamma_{3}>0$ and significant
H3	Technology diffusion J-curve left side	TDI coefficient >0 in mechanism model
H4	Green-technology-oriented NQPs show the most promising reduction potential	$\beta_{1}^{green}<0$, $\beta_{1}^{digital}>0$
H5	NQP total effect dynamic accumulation	Long-term total effect more negative than short-term

**Table 3:** Energy-specific carbon emission accounting parameters.

Energy Type	Unit	NCV	CEF	OX
		(TJ/Unit)	(kg C/TJ)	(%)
Raw coal	10,000 tons	20.908	26.37	0.93 ^*a*^
Crude oil	10,000 tons	41.816	20.08	0.98
Natural gas	10,000 m^3^	38.931	15.32	0.99
Coke	10,000 tons	28.447	29.42	0.93 ^*a*^
Gasoline	10,000 tons	43.070	18.90	0.98
Kerosene	10,000 tons	43.070	19.60	0.98
Diesel	10,000 tons	42.652	20.20	0.98
Fuel oil	10,000 tons	41.816	21.10	0.98
LPG	10,000 tons	50.179	17.20	0.98
Refinery gas	10,000 tons	45.998	18.20	0.98
Other petroleum products	10,000 tons	41.031	20.00	0.98
Other coking products	10,000 tons	28.447	29.42	0.93 ^*a*^
Heat	$10^{4}$ million kJ	0.0341	26.60	0.95
Electricity	100 million kWh	—	—	Grid-specific factor ^*b*^

^*a*^ OX = 0.93 follows the China-specific parameter adopted in provincial carbon accounting studies (e.g., [38]; consistent with the *China Energy Statistical Yearbook* default parameters), while the IPCC (2006) [19] default is 0.98 for coal products. ^*b*^ National average grid emission factor published by the National Development and Reform Commission, declining from 0.7856 kgCO_2_/kWh in 2012 to 0.5568 kgCO_2_/kWh in 2021.

**Table 4:** Variable definitions and descriptive statistics.

Symbol	Variable Name	Definition	Expected Sign (Direct/Indirect)	Data Source
CEI	Carbon emission intensity	CO_2_ emissions/GDP (ton/$10^{4}$ CNY)	n.a.	IPCC method; *China Energy Statistical Yearbook*
NQP Index	New quality productive forces index	Comprehensive index synthesized by entropy-weighted TOPSIS based on 14 indicators	−/− (insignificant)	Calculated by authors
TDI	Technology Diffusion Index	Technology market turnover/regional GDP	+/+	*China Science and Technology Statistical Yearbook*
ISU	Industrial structure upgrading	Tertiary-industry added value/secondary-industry added value	−/−	*China Statistical Yearbook*
ESC	Energy structure optimization	Clean energy generation/total power generation	−/−	*China Energy Statistical Yearbook*
HC	Human capital	Number of college students/total population	−/−	*China Statistical Yearbook*
lnPGDP	Economic development level	Natural logarithm of per-capita GDP ($10^{4}$ CNY/person)	−/+	*China Statistical Yearbook*
lnPGDP^2^	Squared per-capita GDP	Square of natural logarithm of per-capita GDP	+/+	Calculated
URB	Urbanization level	Urban population/total population	−/−	*China Statistical Yearbook*
GOV	Government intervention	Fiscal expenditure/regional GDP	+/+	*China Statistical Yearbook*
OPEN	Openness	Total import and export volume/regional GDP	+/−	*China Statistical Yearbook*

Note: “+” means increasing carbon emissions; “−” means reducing carbon emissions; “n.a.” means not applicable. For NQPs, the expected sign is negative but hypothesized to be statistically insignificant (H1). Per-capita GDP is converted into the unit of ten thousand CNY per person and processed by natural logarithm. $\ln{\mathrm{PGDP}}^{2}$ denotes the square of the natural logarithm of per-capita GDP.

**Table 5:** First-stage diagnostic statistics for the spatial lag term.

Diagnostic Test	Value
First-stage *F*-statistic	26.91
Stock–Yogo 10% maximal IV size critical value ($k_{1}=1,n_{iv}=2$)	19.93
*Conclusion:* Reject weak instruments (F=26.91>19.93)	
Hansen J test *p*-value	0.1599
Significance level α	0.05
*Conclusion:* Cannot reject instrument exogeneity (p=0.1599>0.05)	

**Table 6:** Sample exclusion criteria.

Excluded Region	Reason	Specific Data Issue
Xizang (Tibet)	Missing rate for energy consumption data > 30%	Multiple years lack complete energy balance tables; scattered coal consumption not systematically recorded
Hong Kong SAR	Difference in statistical reporting standards	Uses the IEA accounting framework, inconsistent with mainland China’s IPCC framework
Macao SAR	Statistical reporting standard difference	Same as above
Taiwan Province	Data not available	Not included in the *China Energy Statistical Yearbook*

**Table 7:** Data sources and availability.

Data Type	Source	Access Path (Latest Published)
Energy consumption	*China Energy Statistical Yearbook*	National Bureau of Statistics/CNKI; 2022 ed. (contains 2021 data)
Economic indicators	*China Statistical Yearbook*	https://www.stats.gov.cn/sj/ndsj/ (accessed on 16 May 2026); 2022 ed.
Science & technology	*China Science and Technology Statistical Yearbook*	National Bureau of Statistics; 2022 ed.
Environmental data	*China Environmental Statistical Yearbook*	Ministry of Ecology and Environment; 2022 ed.
Provincial data	Provincial statistical yearbooks	Provincial statistical bureaus/CNKI; 2021–2022 ed. (varies)

**Table 8:** Correspondence between research design and research questions.

Research Question	Design Elements	Test Objectives
RQ1: Spatial asymmetry	DSDM effect decomposition (direct/indirect/total effect)	Test H1 and H5
RQ2: Phased paradox	Add mediating variables into DSDM and compare coefficient changes	Test H2 and H3
RQ3: Heterogeneity	Regional dummy variables, classification of NQP types	Test H4

**Table 9:** Model diagnostic tests and critical values.

Test Type	Statistic	Critical Value	Conclusion (Decision)
Weak instruments (Stock–Yogo)	F=26.91	19.93 ($k_{1}=1,n_{iv}=2$, 10%)	F>19.93 ⇒ reject weak IV
Over-identification (Hansen J)	p=0.1599	α=0.05	p>0.05 ⇒ IV exogenous
Spatial error (LM)	LM = 49.3344	$\chi_{0.05}^{2}\left(1\right)=3.84$	LM > 3.84 ⇒ spatial error exists
SDM reducibility (Wald)	Wald = 25.6780	$\chi_{0.05}^{2}\left(3\right)=7.81$	Wald > 7.81 ⇒ SDM not reducible
Hausman (FE vs. RE)	$\chi^{2}=29.5430$	$\chi_{0.05}^{2}\left(5\right)=11.07$	$\chi^{2}>$ 11.07 ⇒ FE preferred

**Table 10:** DSDM effect decomposition results.

Variable	Short-Term			Long-Term		
	Direct	Indirect	Total	Direct	Indirect	Total
NQPs	0.0072	−0.0100	−0.0028	0.0071	−0.0105	−0.0034
GOV	0.0293 **	−0.1210 ***	−0.0917 ***	0.0281 **	−0.1375 ***	−0.1094 ***
lnPGDP	−0.0060	0.0214	0.0155	−0.0058	0.0242 *	0.0184
lnPGDP^2^	0.0029	0.0300 **	0.0328 **	0.0033	0.0359 **	0.0392 **
OPEN	−0.0019	0.0131	0.0112	−0.0018	0.0151	0.0133
URB	−0.0007	−0.0084 *	−0.0090 *	−0.0008	−0.0100 *	−0.0108 *

Note: *** p<0.01, ** p<0.05, * p<0.1; no asterisk indicates p>0.1.

**Table 11:** Mechanism test: coefficients and effect comparison.

Model Setting	NQP	NQP	Mech.	Short-Term	Long-Term
	Coef.	*p*-Val	Var.	Total Eff.	Total Eff.
Baseline (No Mechanism)	0.0077	0.160	—	—	—
Technology Diffusion (TDI)	0.0063	0.234	0.0021	+0.0553	+0.0670
Industrial Structure (ISU)	0.0120 ^†^	0.092	0.0119	+0.0452	+0.0501
Energy Structure (ESC)	0.0084	0.131	0.0000	+0.0008	+0.0009
Human Capital (HC)	0.0073	0.187	−0.0008	−0.0191	−0.0227

Note: ^†^ denotes marginal significance (p<0.1).

**Table 12:** Regional heterogeneity: total effect and inter-group difference.

Region	Total Effect	Difference vs. Western Region	*p*-Value
Western (Benchmark)	0.0485 **	—	—
Eastern	0.0053	$-0.0432^{\;†}$	0.0553
Central	0.0343	−0.0142	0.5892

Note: ** p<0.05, ^†^ p<0.1.

**Table 13:** NQP type classification rules.

Type	Indicators Included	Core Attribute	Entropy Weight
Digital technology	5G mobile users, mobile internet traffic, and software business income	Information infrastructure & data processing	0.5566 0.2215 0.2218
Green technology	New energy power share, energy per unit GDP, and pollution control investment	Energy structure optimization & environmental governance	0.3835 0.0919 0.5246
High-tech industry	High-tech employees share, industrial robot penetration, integrated circuit output, R&D personnel, and high-tech patents granted	Technology R&D & advanced manufacturing	0.0711 0.1847 0.3640 0.2128 0.1675

Note: Three indicators (education structure, new materials output, and informatization integration) are excluded from the type indices due to mixed attributes or insufficient direct environmental relevance, but they remain in the benchmark NQP index.

**Table 14:** DSDM estimation results for NQP types.

Type	Coefficient	Std. Error	*p*-Value	95% CI	Short-Term Total
Digital technology	0.0015	0.0048	0.763	[−0.0080,0.0109]	−0.0385
Green technology	−0.0009	0.0052	0.858	[−0.0111,0.0093]	−0.0113
High-tech industry	0.0105	0.0036	0.004	[0.0034,0.0176]	0.0397

Note: Standard errors and confidence intervals are based on robust standard errors. The dependent variable is carbon emission intensity (CEI). All models include the same set of control variables (GOV, lnPGDP, lnPGDP^2^, OPEN, URB) and are estimated using the DSDM framework with IV-GMM.

**Table 15:** Summary of hypothesis validation results.

Hyp.	Core Proposition	Empirical Result	Status	Key Evidence
H1	Direct effect insignificant; spatial spillover limited	Direct 0.0072 (p>0.1); indirect −0.0100 (p>0.1)	Supported	Table 10
H2	Spatial EKC positive intensification	lnPGDP^2^ spatial indirect 0.0300 ** (p<0.05); marginal effect 0.084–0.168	Supported	Table 10, Figure 1
H3	Technology diffusion J-curve left side	TDI short-term total 0.0553; inflection point not reached	Tentatively supported ^*a*^	Table 11
H4	Green-tech NQPs: effective reducer	Green type: negative (insignificant); digital type: carbon rebound	Partially supported ^*b*^	Section 4.5.2
H5	NQP total effect dynamic accumulation	Short-term −0.0028 vs. long-term −0.0034 (both insignificant)	Not supported	Table 10

^*a*^ Interpretation is tentative due to TDI’s inability to distinguish green from general technology transactions. ^*b*^ Partial support reflects that green-technology NQPs show the expected negative direction but are not statistically significant at conventional levels in the disaggregated DSDM estimation. ** denotes statistical significance at the 5% level.

## Data Availability

The raw data analyzed in this study are derived from publicly available official statistical yearbooks in China, including the *China Statistical Yearbook* (https://www.stats.gov.cn/sj/ndsj/ (accessed on 16 May 2026)), *China Energy Statistical Yearbook*, and *China Science and Technology Statistical Yearbook*. The processed panel data and NQP index calculations are available from the corresponding author upon reasonable request.

## Abbreviations

The following abbreviations are used in this manuscript:

NQPs	New quality productive forces
CEI	Carbon emission intensity
EKC	Environmental Kuznets curve
SDM	Spatial Durbin model
DSDM	Dynamic spatial Durbin model
SLM	Spatial lag model
SEM	Spatial error model
IV-GMM	Instrumental variable generalized method of moments
GDP	Gross domestic product
PGDP	Per-capita GDP
TDI	Technology diffusion index
ISU	Industrial structure upgrading
ESC	Energy structure optimization
HC	Human capital
GOV	Government intervention
OPEN	Openness
URB	Urbanization
LISA	Local indicators of spatial association
5G	Fifth-generation mobile networks
OECD	Organisation for Economic Co-operation and Development
IPCC	Intergovernmental Panel on Climate Change
AHP	Analytic hierarchy process
TOPSIS	Technique for order preference by similarity to ideal solution

## Appendix A. Descriptive Statistics

**Table A1:** Descriptive statistics.

Variable	Obs.	Mean	Std. Dev.	Min	Max
CEI	300	1.847	1.023	0.312	5.467
NQP	300	0.312	0.156	0.089	0.876
TDI	300	0.015	0.012	0.001	0.089
ISU	300	1.234	0.567	0.456	3.210
ESC	300	0.189	0.134	0.012	0.678
HC	300	0.023	0.008	0.009	0.056
lnPGDP	300	1.673	0.405	0.679	2.854
URB	300	0.589	0.123	0.312	0.896
GOV	300	0.234	0.089	0.123	0.567
OPEN	300	0.312	0.456	0.023	2.345

## Appendix B. NQP Index System and Weights

**Table A2:** NQP indicators and weights (benchmark model).

Dimension	Category	Indicator	Sub-Indicator	Weight
Material Elements	Labor	New labor quantity	High-tech employees share	0.0272
		New labor structure	Education structure	0.0814
	Means of labor	New production tools	Industrial robot penetration	0.0707
			Integrated circuit output	0.1393
		New infrastructure	5G mobile user indicator	0.2106
	Objects of labor	New energy	New energy power share	0.0248
		New materials	New materials output	0.0608
		Low-carbon productivity	Energy per unit GDP	0.0321
Permeating Elements	New technology	Technology R&D	R&D in high-tech industries	0.0558
		Innovation output	High-tech patents granted	0.0641
	Data elements	Big data generation	Mobile internet traffic	0.0838
		Big data processing	Software business income	0.0839
	Production organization	Greening	Pollution control investment	0.0339
		Integration	Informatization integration	0.0314

**Table A3:** NQP indicators and weights (excluding the 5G mobile user indicator).

Dimension	Category	Indicator	Sub-Indicator	Weight
Material Elements	Labor	New labor quantity	High-tech employees share	0.0345
		New labor structure	Education structure	0.0814
	Means of labor	New production tools	Industrial robot penetration	0.0895
			Integrated circuit output	0.1765
	Objects of labor	New energy	New energy power share	0.0314
		New materials	New materials output	0.0771
		Low-carbon productivity	Energy per unit GDP	0.0406
Permeating Elements	New technology	Technology R&D	R&D in high-tech industries	0.0707
		Innovation output	High-tech patents granted	0.0812
	Data elements	Big data generation	Mobile internet traffic	0.1062
		Big data processing	Software business income	0.1063
	Production organization	Greening	Pollution control investment	0.0430
		Integration	Informatization integration	0.0398

## Appendix C. Spatial Weight Matrix Comparison

**Table A4:** Comparison of three spatial weight matrices.

Indicator	Economic Distance	Geographic Adjacency	Inverse Geo. Distance
Spatial lag coef. ρ	0.7097 **	0.1810	−2.4024
Standard error	0.2954	0.4264	1.7408
*p*-value	0.0246	0.671	0.168
First-stage *F*	25.46	21.63	23.54
Hansen *J p*-value	0.2073	0.0169	0.4003
$R^{2}$	0.562	0.226	0.376
Model validity	Valid	IV invalid	Unreasonable

Note: ** p<0.05.

## Appendix D. Mechanism Test Full Results

**Table A5:** Key diagnostic parameters of each mechanism model.

Model	First-Stage *F*	Spatial ρ	ρ *p*-Val	Hansen *J p*-Val
Baseline	26.91	0.6164	0.0370	0.1599
TDI	23.98	0.6434	0.0248	0.1626
ISU	26.41	0.5077	0.0905	0.1514
ESC	23.87	0.4969	0.0994	0.1094
HC	24.65	0.6236	0.0366	0.1502

**Table A6:** Mechanism test: full coefficient summary.

Model	NQP		M		WX_M	
	Coef.	*p*	Coef.	*p*	Coef.	*p*
Baseline	0.0077	0.160	—	—	—	—
TDI	0.0063	0.234	0.0021	0.705	0.0176	0.2043
ISU	0.0120 ^†^	0.092	0.0119	0.159	0.0103	0.7140
ESC	0.0084	0.131	0.0000	0.766	0.0004	0.0582
HC	0.0073	0.187	−0.0008	0.851	−0.0064	0.2311

Note: ^†^ p<0.1. Model diagnostic parameters (first-stage *F*, spatial ρ, Hansen *J*) are reported in Table A5.

**Table A7:** Effect decomposition of mechanism variables.

Variable	Short-Term			Long-Term		
	Direct	Indirect	Total	Direct	Indirect	Total
TDI	0.0051	0.0502	0.0553	0.0059	0.0612	0.0670
ISU	0.0136	0.0315	0.0452	0.0140	0.0361	0.0501
ESC	0.0001	0.0008	0.0008	0.0001	0.0008	0.0009
HC	−0.0018	−0.0173	−0.0191	−0.0021	−0.0207	−0.0227

## Appendix E. Robustness Checks Detailed Results

**Table A8:** Sensitivity of DSDM estimates to outliers: baseline vs. 1–99% winsorized sample.

Variable	Full Sample (Baseline)		Winsorized	
	Coef.	*p*-Val	Coef.	*p*-Val
GOV	0.0365	0.0040	0.0376	0.0030
WX_GOV	−0.0717	0.0050	−0.0717	0.0050
WX_lnPGDP^2^	0.0115	0.0140	0.0109	0.0150
WX_URB	−0.0033	0.0320	−0.0031	0.0370
ρ	0.6164	0.0370	0.7097	0.0250
First-stage *F*	26.9100	—	25.4600	—
Hansen *J p*-val	—	0.1599	—	0.2070

Note: ρ is the spatial autoregressive coefficient of the dependent variable. First-stage *F* and Hansen *J* are diagnostic statistics for instrumental variable validity.

**Table A9:** Results after excluding municipalities.

Variable	Full Sample (Baseline)		Excl. Municipalities	
	Coef.	*p*-Val	Coef.	*p*-Val
GOV	0.0365	0.0040	0.0376	0.0050
WX_GOV	−0.0717	0.0050	−0.0868	0.0340
WX_lnPGDP^2^	0.0115	0.0140	0.0181	0.0300
WX_URB	−0.0033	0.0320	−0.0040	0.0720
ρ	0.6164	0.0370	0.3327	0.3520
First-stage *F*	26.9100	—	19.3300	—
Hansen *J p*-val	—	0.1599	—	0.1780
Observations	300.0000	—	260.0000	—

Note: ρ is the spatial autoregressive coefficient of the dependent variable. First-stage *F* and Hansen *J* are diagnostic statistics for instrumental variable validity.

**Table A10:** DSDM results (excluding the 5G mobile user indicator).

Effect Type	With the 5G Mobile User Indicator	Excluding the 5G Mobile User Indicator
Short-term direct	0.0072 (insignificant)	0.0135 ** (p=0.003)
Short-term indirect	−0.0100	+0.0269
Short-term total	−0.0028	+0.0404
Spatial lag coef. ρ	0.6164 **	0.6976 **

Note: ** p<0.05.

## Appendix F. Additional Sensitivity Analysis: The Role of 5G Indicator Weighting

### Appendix F.1. The Role of 5G Indicator Weighting

To assess whether the entropy-TOPSIS weighting scheme drives the main findings, we examined the sensitivity of the results to the 5G mobile user indicator, which receives the largest single weight (21.06%) due to its structural jump from zero before its commercialization in 2019. Table A11 compares the NQP effect decomposition under the benchmark specification and the specification excluding the 5G indicator.

**Table A11:** Comparison of NQP effect decomposition under alternative indicator sets.

Indicator Set	NQP Direct	NQP Indirect	NQP Total	ρ
Benchmark (all 14 indicators)	0.0072	−0.0100	−0.0028	0.6164 **
Excluding 5G users	0.0135 **	+0.0269	+0.0404	0.6976 **

Note: ** p<0.05. Complete decomposition results for all variables are reported in Table 10.

The results reveal a notable sensitivity: excluding the 5G indicator reverses the indirect effect from negative (−0.0100) to positive (+0.0269). This suggests that the benchmark model’s “negative spatial spillover” conclusion under the benchmark model is partially driven by the dominant weight assigned to the 5G indicator (21.06%). After excluding 5G, the digital-technology-type indicators (integrated circuit output, mobile internet traffic, and software revenue) still account for 38.9% of the total weight, while green-technology-type indicators (new energy share, energy intensity, and pollution control investment) account for 11.50%, up from 9.08% in the benchmark specification. This modest increase confirms that the NQP index remains digital-heavy even without the 5G indicator.

The spatial dependence of carbon emissions (ρ) remains significant under both specifications (0.6164** and 0.6976**), confirming the robustness of the spatial correlation findings.

### Appendix F.2. Limitations and Future Methodological Directions

We acknowledge that alternative weighting methods—such as the analytic hierarchy process (AHP), principal component analysis (PCA), or machine-learning-based feature weighting—could provide additional validation of our findings. However, these methods require fundamentally different assumptions (e.g., expert judgment for AHP; orthogonality assumptions for PCA) and are beyond the scope of the current study. We have identified this as a key direction for future research in Section 6, where we suggest that future studies could combine subjective and objective weighting approaches to construct a more stable and green-oriented NQP evaluation system.

## References

[1] Han W., Zhang R., Zhao F. (2024). Measurement of New Quality Productive Forces and New Drivers of China’s Economic Growth. J. Quant. Tech. Econ..

[2] Shen L., Geng C., Li X., Dong J. (2025). Research on the Mechanism and Path of Green Energy Efficiency Improvement Empowered by New Quality Productive Forces. Manag. Rev..

[3] Anselin L. (1988). Spatial Econometrics: Methods and Models.

[4] Liu Y., Xiao H., Zikhali P., Lv Y. (2014). Carbon Emissions in China: A Spatial Econometric Analysis at the Regional Level. Sustainability.

[5] Li L., Xia Z., Yi J., Qi R., Cheng J. (2024). Industrial Agglomeration and PM_2.5_ Pollution in Yangtze River Economic Belt in China: Non-linear Estimation and Mechanism Analysis. Front. Environ. Sci..

[6] Zhou S., Jiang Q. (2020). Spatial Effect of Regional Carbon Emission Intensity in China Based on Dynamic SDM. J. Hunan Univ. Soc. Sci..

[7] Han X.F., Chen L.T., Song W.F., Li B.X., Chen X.Y. (2023). Dynamic Adjustment Mechanism of Carbon Emissions on Industrial Upgrading Driven by Green Technology Innovation. China Popul. Resour. Environ..

[8] Sorrell S. (2009). Jevons’ Paradox Revisited: The Evidence for Backfire from Improved Energy Efficiency. Energy Policy.

[9] Wang Z., Mae M., Nishimura S., Matsuhashi R. (2024). Vehicular Fuel Consumption and CO_2_ Emission Estimation Model Integrating Novel Driving Behavior Data Using Machine Learning. Energies.

[10] Wang Z., Matsuhashi R. (2026). Low-energy miniature wearable air-conditioner with direct cold-air delivery: A novel water–electricity hybrid energy system. Build. Environ..

[11] Grossman G.M., Krueger A.B. (1995). Economic Growth and the Environment. Q. J. Econ..

[12] Dinda S. (2004). Environmental Kuznets Curve Hypothesis: A Survey. Ecol. Econ..

[13] Stern D.I. (2004). The Rise and Fall of the Environmental Kuznets Curve. World Dev..

[14] Copeland B.R., Taylor M.S. (2004). Trade, Growth, and the Environment. J. Econ. Lit..

[15] Liu Z.B. (2024). New Production Relations Driven by New Quality Productive Forces: Trends, Challenges and Countermeasures. Financ. Trade Econ..

[16] OECD (2011). Towards Green Growth.

[17] Zhao T., Zhang Z., Liang S.K. (2020). Digital Economy, Entrepreneurial Activity and High-Quality Development: Empirical Evidence from Chinese Cities. Manag. World.

[18] Chen J.H., Chen Y., Chen M.M. (2020). China’s High-Quality Economic Development Level, Regional Differences and Dynamic Evolution of Distribution. J. Quant. Tech. Econ..

[19] IPCC (2006). 2006 IPCC Guidelines for National Greenhouse Gas Inventories. Prepared by the National Greenhouse Gas Inventories Programme. https://www.ipcc-nggip.iges.or.jp/public/2006gl/.

[20] Lin B.Q., Liu X.Y. (2010). China’s Urbanization Stage and Carbon Emissions: Influencing Factors and Emission Reduction Strategies. Econ. Res. J..

[21] LeSage J.P., Pace R.K. (2009). Introduction to Spatial Econometrics.

[22] LeSage J.P., Fischer M.M. (2008). Spatial Growth Regressions: Model Specification, Estimation and Interpretation. Spat. Econ. Anal..

[23] Elhorst J.P. (2010). Applied Spatial Econometrics: Raising the Bar. Spat. Econ. Anal..

[24] Wang J.K., Han Q. (2021). Spatial-temporal Pattern of the Coupling Coordination of Urban Economy-Society-Environment in China. Econ. Geogr..

[25] Griliches Z. (1957). Hybrid Corn: An Exploration in the Economics of Technological Change. Econometrica.

[26] Rennings K. (2000). Redefining Innovation—Eco-Innovation Research and the Contribution from Ecological Economics. Ecol. Econ..

[27] Rogers E.M. (2003). Diffusion of Innovations.

[28] Acemoglu D., Aghion P., Bursztyn L., Hemous D. (2012). The Environment and Directed Technical Change. Am. Econ. Rev..

[29] Grubb M. (2004). Technology Innovation and Climate Change Policy: An Overview of Issues and Options. Keio Econ. Stud..

[30] Aghion P., Dechezleprêtre A., Hemous D., Martin R., Van Reenen J. (2016). Carbon Taxes, Path Dependency, and Directed Technical Change: Evidence from the Auto Industry. J. Political Econ..

[31] Porter M.E., van der Linde C. (1995). Toward a New Conception of the Environment-Competitiveness Relationship. J. Econ. Perspect..

[32] Ambec S., Cohen M.A., Elgie S., Lanoie P. (2013). The Porter Hypothesis at 20: Can Environmental Regulation Enhance Innovation and Competitiveness?. Rev. Environ. Econ. Policy.

[33] Dechezleprêtre A., Sato M. (2017). The Impacts of Environmental Regulations on Competitiveness. Rev. Environ. Econ. Policy.

[34] Arthur W.B. (1989). Competing Technologies, Increasing Returns, and Lock-In by Historical Events. Econ. J..

[35] Unruh G.C. (2000). Understanding Carbon Lock-in. Energy Policy.

[36] Seto K.C., Davis S.J., Mitchell R.B., Stokes E.C., Unruh G., Ürge-Vorsatz D. (2016). Carbon Lock-In: Types, Causes, and Policy Implications. Annu. Rev. Environ. Resour..

[37] Erickson P., Kartha S., Lazarus M., Tempest K. (2015). Assessing Carbon Lock-in. Environ. Res. Lett..

[38] Shan Y., Guan D., Zheng H., Ou J., Li Y., Meng J., Mi Z., Liu Z., Zhang Q. (2018). China CO_2_ Emission Accounts 1997–2015. Sci. Data.

[39] Zhang Y.J. (2011). The Impact of Financial Development on Carbon Emissions: An Empirical Analysis in China. Energy Policy.

[40] Hao Y., Chen H.Q., Zhang Q.M. (2016). Will Income Inequality Affect Environmental Quality? Analysis Based on China’s Provincial Panel Data. Ecol. Indic..

[41] Elhorst J.P. (2014). Spatial Econometrics: From Cross-Sectional Data to Spatial Panels.

[42] Stock J.H., Yogo M., Andrews D.W.K., Stock J.H. (2005). Testing for Weak Instruments in Linear IV Regression. Identification and Inference for Econometric Models: Essays in Honor of Thomas Rothenberg.

[43] Kelejian H.H., Prucha I.R. (1998). A Generalized Spatial Two-Stage Least Squares Procedure for Estimating a Spatial Autoregressive Model with Autoregressive Disturbances. J. Real Estate Financ. Econ..

[44] Lee L.F., Yu J. (2010). Estimation of Spatial Autoregressive Panel Data Models with Fixed Effects. J. Econom..

[45] Blundell R., Bond S. (1998). Initial Conditions and Moment Restrictions in Dynamic Panel Data Models. J. Econom..

[46] National Bureau of Statistics of China (2011). Rules for the Compilation of Statistical Zoning Codes and Urban-Rural Classification Codes. http://www.stats.gov.cn/sj/tjbz/gjtjbz/202302/t20230213_1902741.html.

